# Eros, Beauty, and Phon-Aesthetic Judgements of Language Sound. We Like It Flat and Fast, but Not Melodious. Comparing Phonetic and Acoustic Features of 16 European Languages

**DOI:** 10.3389/fnhum.2021.578594

**Published:** 2021-02-23

**Authors:** Vita V. Kogan, Susanne M. Reiterer

**Affiliations:** ^1^School of European Culture and Languages, University of Kent, Kent, United Kingdom; ^2^Department of Linguistics, University of Vienna, Vienna, Austria; ^3^Teacher Education Centre, University of Vienna, Vienna, Austria

**Keywords:** phon-aesthetics, language attitudes and ideologies, speech melody, speech rate, language perception, crosslinguistic comparison, rhythm in language, prosody and intonation perception

## Abstract

This article concerns sound aesthetic preferences for European foreign languages. We investigated the phonetic-acoustic dimension of the linguistic aesthetic pleasure to describe the “music” found in European languages. The Romance languages, French, Italian, and Spanish, take a lead when people talk about melodious language – the music-like effects in the language (a.k.a., phonetic chill). On the other end of the melodiousness spectrum are German and Arabic that are often considered sounding harsh and un-attractive. Despite the public interest, limited research has been conducted on the topic of phonaesthetics, i.e., the subfield of phonetics that is concerned with the aesthetic properties of speech sounds (Crystal, 2008). Our goal is to fill the existing research gap by identifying the acoustic features that drive the auditory perception of language sound beauty. What is so music-like in the language that makes people say “it is music in my ears”? We had 45 central European participants listening to 16 auditorily presented European languages and rating each language in terms of 22 binary characteristics (e.g., beautiful – ugly and funny – boring) plus indicating their language familiarities, L2 backgrounds, speaker voice liking, demographics, and musicality levels. Findings revealed that all factors in complex interplay explain a certain percentage of variance: familiarity and expertise in foreign languages, speaker voice characteristics, phonetic complexity, musical acoustic properties, and finally musical expertise of the listener. The most important discovery was the trade-off between speech tempo and so-called linguistic melody (pitch variance): the faster the language, the flatter/more atonal it is in terms of the pitch (speech melody), making it highly appealing acoustically (sounding beautiful and sexy), but not so melodious in a “musical” sense.

## Introduction

There is almost universal agreement that Italian, Spanish, and French are appealing and melodious languages to the human ear. Italian, it is often said, is the language of opera, and only a rare singer does not have it in their linguistic repertoire. There are several reasons why Italian might be so pleasant to hear. Dr. Patti Adank, professor of Speech, Hearing, and Phonetic Science at the University College London, says that an open syllabic structure and a high vocalic share make Italian the optimal language for singing (as cited in Kerr, 2017). Matteo Dalle Fratte, a musicologist and founder of *Melofonetica.com*, says that “Italian is the language built to be sung,” that the alternation of short and long consonants in Italian (gemination or double consonants) produces “the agogic accent” and “incredible expression and dramatic tension to the text” (Dalle Fratte, 2018). Opera lovers seem to agree. Online forums for singers are replete with comments such as “As for the best sounding language when sung, I feel that it’s [*sic*] Finnish, and Italian and Spanish sound good too. All are heavily vowel-y languages which is pretty much essential for a good singing language” (Guest123456, 2010).

Whereas the Romance languages are frequently described as melodious, beautiful and sexy, German and Arabic, on the other side of the likeability spectrum, are for some too harsh or vocally unpleasant due to their consonant clusters, and to many Western ears, tonal languages, such as Cantonese or Mandarin, sound whiny (Science Chat Forum, 2011; Quora, 2015). Certainly, it is hard to separate the effects of phonetic features from the influence of the languages’ socio-cultural aura – the speakers and the history behind a language. French might sound lovely to one’s ear because it has a high vocalic index (every other sound in French is a vowel) but also because listening to French brings memories of Les Champs-Élysées, fragrant wines, and Duma’s novels. In our previous study (Reiterer et al., 2020), we found that pre-existing socio-cultural factors, like, second language experience, as well as the speaker’s voice, explained most (two thirds) of the variation in listeners’ aesthetic judgments. Yet, the phonetic properties of languages also played a significant role. In the present study, we focus on the phonetic-acoustic dimension of the linguistic aesthetic pleasure and try to quantify the “music” found in European languages. Despite the public interest, there has been little research into phonaesthetics, a subfield of phonetics that is concerned with the aesthetic properties of speech sounds (Crystal, 2008). This is surprising, given the success of aesthetic research in other fields: e.g., the aesthetics of objects (Jacobsen et al., 2004), the experience of music (Brattico et al., 2013; Reuter and Siddiq, 2017), and art (Zaidel et al., 2013; Leder et al., 2014), as well as the appreciation of mathematical beauty (Zeki et al., 2014).

The idea that some languages sound like music is not counterintuitive. According to the *musical protolanguage hypothesis*, speech and music originate from the same source; i.e., they come from the imitation and modification of environmental sounds to express basic emotions such as love, anger, pity, and sadness (Kirby, 2011; Fitch, 2013; Ma et al., 2019). Because of the vocal tract constraints associated with speaking and singing, similar emotions are conveyed by similar acoustic features in both domains. Previous studies have shown that the speech rate or tempo and F0/pitch function more or less the same way in speech and music in terms of their effects on listeners’ ratings (Juslin, 1997, 2000).

Speech rate (tempo in music) is a temporal aspect that typically signifies a certain number of units per duration; e.g., syllables per second. In both speech and music, rate or tempo increases with high-arousal or “active” emotions such as anger, fear, and happiness. The opposite is true for low-arousal or “passive” emotions such as sadness and tenderness (Juslin and Laukka, 2003; Ma and Thompson, 2015). Fundamental frequency F0 (analogous to pitch in music and the acoustic correlate of the main portion of the perceived pitch in the speaker’s voice) is characterized by the rate at which the vocal folds open and close across the glottis. In both music and speech, a low pitch is associated with sadness and a high pitch – with happiness. A rising F0 contour evokes active emotions, whereas a falling F0 contour is associated with passive emotions (Cordes, 2000). The same holds true for pitch variation: happy, angry, and frightened responses increase with higher pitch variation and the perception of sad and angry stimuli is influenced by lower pitch variation (Breitenstein et al., 2001).

Not all studies demonstrate overlapping emotional ratings for speech and music. Ilie and Thompson (2006) manipulated several acoustic cues in both domains, and even though they observed similarities for some of the cues – e.g., fast speech and fast music were perceived as more energetic and tense compared to slower speech and music, – the effects were not consistent across all acoustic dimensions. For example, participants found a high-pitched speech (though not music) more pleasant. The authors concluded that even though the same circuitry might be involved in connecting acoustic events and their corresponding affective meanings in both speech and music, different attentional strategies might be used for the two types of stimuli; that is, listeners pay greater attention to prominent aesthetic properties of music than language, where verbal information is probably the primary attention attractor. Some studies find that speech and music have domain-specific cues to emotions because they have different structural features and functions (e.g., Krumhansl, 1990). For example, Quinto et al. (2013), after measuring pitch variability and rhythmic properties of speech and music, concluded that whereas a changing pitch conveys emotional intentions in speech, it does not behave the same way in music. One explanation for this, they say, relates to cross-cultural differences in emotional verbal communication – the so-called “pull-effects” – (Scherer et al., 2003), and even though emotional decoding across cultures is relatively good, people within a culture are still better able to identify emotions than outsiders (Mesquita, 2003). However, the cultural component, which is inseparable from language and emotional events, makes comparisons between verbal and musical stimuli even more challenging.

It should be noted though, that since there are no direct acoustic analogs between speech and music, it is problematic to make conclusive comparisons. Chow and Brown (2018) attempted to solve this problem by using the musical notation in their analysis of speech melody, converting the fundamental-frequency trajectories of the recorded words and utterances from hertz into semitones and transcribing them into musical scores of a relative pitch. They reported that, compared to music, speech is atonal and characterized by a weak type of chromaticism. Nevertheless, even within the compressed pitch range of standard speech production, language-specific melodic patterns can be found. Font-Rotchés and Torregrosa-Azor (2015) compared the intonation of yes-no questions in Spanish and German using the Melodic Analysis of Speech, a method of analysis that is based on the principle of phonic hierarchy and the measures of the F0 of the tonal segments, the vowels. Although the authors observed a close correspondence between some Spanish and German melodic patterns, they also reported substantial differences in the tonal range: to the point that a statement produced in one language might sound like a question in another language. Mennen et al. (2012) confirmed the existence of significant cross-linguistic differences in the F0 range. Yet, the aesthetic value of the melodic patterns and how they might contribute (or not) to the overall music-like effect in some languages remain unknown.

Although our knowledge of the overlap between music and language is notable (Sammler, 2020), their aesthetic acoustic properties are still poorly understood. In compariosn to speech, the aesthetic investigations of music are more promising. Several studies describe rhythm and its perceptual attribute, the beat, and tonal harmony as the most prominent contributors to auditory pleasure (e.g., Brattico et al., 2013). Rhythm perception arise from a grouping mechanism that creates patterns of prominence recurring in time (Arvaniti, 2009; Falk et al., 2014). While it is quite likely that the aesthetic value of rhythm is shaped by biological constraints – e.g., the limitations of working memory (Ravignani et al., 2016) or even by the inner pacemaker: heart beat (Chahal et al., 2017) – the formal structure of a musical piece is also important: tonality, harmony, and meter create specific temporal regularities, which, when they meet the expectations, build an emotional aesthetic response. In this sense, anticipation or expectancy, which also depends on musical knowledge, is the essential mechanism for a pleasurable experience (Steinbeis et al., 2006; Vuust and Kringelbach, 2010). Some listeners experience “chills,” an intense physical sensation such as goosebumps or trembling, in response to a favorite tune or melody (Reuter and Oehler, 2011; Starcke et al., 2019), and while musical events that elicit such reactions vary from person to person, there are a few patterns that might be connected to chills: the onset of vocals, the beginning of a structurally new part, and contrasting voices are strong acoustic triggers (Grewe et al., 2007; Guhn et al., 2007).

Compared to music, little is known about the phonetic chill or the auditory pleasure that arises from listening to languages. First of all, it is hard to evaluate the aesthetic value of rhythm in language because speech rhythmic patterns are not beat-based (or metrical), unlike the rhythmic patterns of music. Ozernov-Palchik and Patel (2018) observe that “the temporal patterning of linguistic units is highly structured, but is not based on an underlying grid of equal time intervals” (p. 166). Beat-based processing and speech processing may be cognitively related at a more abstract level concerned with prediction in structured sequences: after all, listeners routinely predict upcoming linguistic material, although the prediction is not based on temporal periodicity but rather on phonological, semantic, and syntactic structures. Yet, there is something in the speech that contributes to the perceptual experience of a beat. Infant et al. (2013) suggest that the beat distribution patterns in speech are cued by stressed syllables and p-centers, a psychological phenomenon that coincides with syllabic nuclei and vowel onsets (Lin and Rathcke, 2020). Arvaniti (2012) also connects rhythm with syllabic prominence.

On closer examination, we can see that a more regular syllabic structure (e.g., CVCV, where “C” stands for a consonant and “V” for a vowel) might produce a similar pleasurable anticipatory effect that is fundamental in music (Steinbeis et al., 2006; Vuust and Kringelbach, 2010). For that reason, it might have a higher aesthetic value than a more complex unpredictable syllabic structure (e.g., CVCCVCC). There is, indeed, a language-universal preference for the CV structure that, at times, overrides a preference for native-specific structures (Greenberg, 1965; Blevins, 1995). Either way, it is reasonable to assume that languages that predominantly use the CV structure also sound more pleasant. Unlike Italian, which often uses the CV structure, German, its northern neighbor, displays a structural variety with complex syllabic combinations, including heavy consonant clusters (Rabanus, 2003).

Another reason for the difficulty in quantifying the timing characteristics of the linguistic rhythm is that it is not a unidimensional phenomenon and influenced by several factors, including F0 movements (Tilsen and Arvaniti, 2013). So, unlike in the case of music, the linguistic rhythm is the product of various phonological phenomena, each interacting with others. It does not mean that linguistic rhythm is unconnected to musical rhythm: several studies have shown the influence of speech rhythms on non-linguistic rhythmic grouping preferences (e.g., when the composer’s native language influences the composition) (Patel et al., 2006; Jekiel, 2014).

Remarkably few empirical studies have investigated the music-like effects in language. Even though much is known about the intonation and the rhythmic architecture of speech, it is still unclear what aesthetic value these elements have and how they contribute (or not) to a pleasurable auditory experience derived from listening to the spoken word. While prior studies compared the sounds of language and music (Patel et al., 2006; Chow and Brown, 2018), none looked at the aesthetic value of the acoustic parameters responsible for music-like effects in language. In this regard, the goal of the present study is *multi-*disciplinary (as opposed to just inter-disciplinary) since we aim at quantifying a phenomenon at the juncture of three domains: linguistics/language sciences, musicology, and aesthetics. Although this approach is advantageous in many senses as it allows for new questions to be formed in a new way, it also has a number of limitations. For example, we are not aware of the previous research that would employ a similar design and, therefore, provide important guidance for our methodology and data analysis. Thus, we had to rely solely on our own knowledge of the question and scientific intuition, which makes the present study highly exploratory in nature. Another challenge for any inter- and multidisciplinary research is developing a common language across the disciplines to describe a complex phenomenon in the most comprehensive way. Despite these and other limitations, we believe it is important to begin a conversation on this topic and open the line of research that future scholars can confirm, refute, or finesse at their leisure.

Our immediate goal is to fill an existing gap in the research by identifying those acoustic features that drive the auditory perception of language beauty to create a musical effect in the sound of the language. What is so musical in a language that makes people say “It’s music in my ears”? Why are we so mesmerized by the sounds of the Romance languages, especially Italian, French and Spanish (the Latin lover effect), and less enthusiastic about the Germanic and Slavic families? Is it true that Italian is a language built to be sung? Can we explain at least part of the charm or ‘sound pleasure’ (“Ohrenschmaus”) by characteristics derived from acoustic-phonetic measures? Here, we are primarily interested in the auditory allure of the Romance languages, which have been consistently marked across various surveys as the most melodious languages in the world (Burchette, 2014). In our previous study (Reiterer et al., 2020), the Romance languages were described as “pleasant to listen to,” “melodic,” and the “languages of music and songs” (e.g., one participant observed: “French sounds to me very soft and “round.” It is often the language of love and in many songs, some phrases are in French. Lady Gaga: Bad Romance/Christina Aguilera: Lady Marmalade/ABBA: Voulez-vous”). However, here we look at the acoustic parameters that are responsible for pleasurable aesthetic effects in music – rhythm and melody – and explore the higher-order linguistic phenomena we seem to perceive, the phonetic chill. This is the first in a series of studies that, like any step into the unknown, is highly exploratory. For this reason, it is peppered with caveats and limitations that we discuss at the end of this article.

To summarize, our research questions are:

1. What acoustic-phonetic features are responsible for the music-like or phon-aesthetically pleasing effects in languages?
2. How are these features distributed across language families? Do the Romance languages lead the list in this sense?

Apart from shedding light, we hope, on the nature of the hedonic pleasure derived from the architecture of language sounds, this study has pedagogical implications for foreign language learning. Appreciating the acoustic makeup of a target language might activate additional affective learning pathways in the learner’s brain and support auditory memory. For example, neuropsychological studies show that emotional events are remembered better than neutral events, thanks to the amygdalae – two almond-shaped nuclei in the brain that enhance the function of the medial temporal lobe memory system (Dolcos et al., 2004; Koelsch et al., 2006). Approaching language as a song also helps to alleviate speaking anxiety and produces an overall relaxing effect that is essential for successful learning (Fonseca-Mora et al., 2011]BR110). Teachers can use the acoustic properties of the language-to-be-learned and complement the classroom work with synesthetic activities that emphasize specific phonetic features (Wrembel, 2010).

## Materials and Methods

### Participants

The participants (*N* = 45) were students or young academics with the following native languages (L1s = first languages): Slovenian (22), German (11), English (5), Serbo-Croatian (3), Finish (1), Italian (1), Kazak (1), and Portuguese (1). Participants’ ages ranged between 22 and 49. On average, the participants reported being able to speak 2.9 foreign languages (*SD* = 1.6, min = 0, max = 8). The following languages were mentioned as participants’ foreign languages or L2s (from most to least common): English (71%), German, Italian, French, Croatian, and Russian. Other languages mentioned as L3/LX (in the alphabetic order): Arabic, Chinese, Esperanto, Hungarian, Finnish, Japanese, Ladin, Latin, Portuguese, Romanian, Russian, Slovene, Spanish, Swedish, Turkish, and Welsh.

### Materials

**FIGURE 1:**
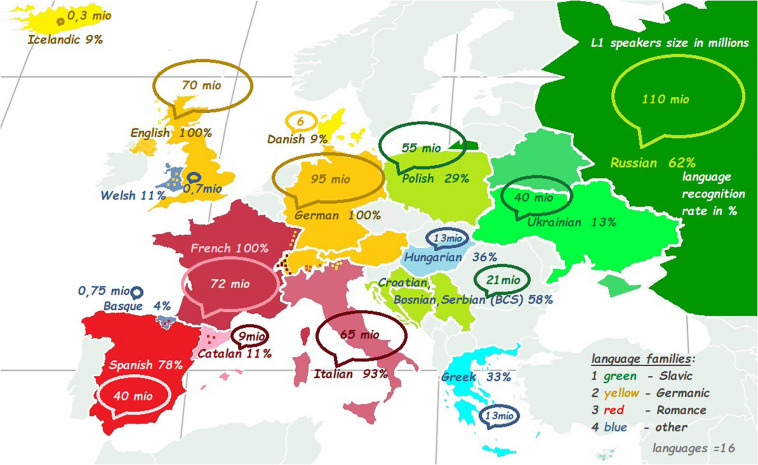
The number of speakers in millions and the recognition rate in percentage. The green color refers to Slavic languages, yellow – Germanic languages, red – Romance languages, and blue indicates other language families or languages-isolates.

The recordings of the 16 European languages were presented as auditory stimuli recorded by native speakers, half of which were females (Supplementary Table 2S). Each stimulus featured a reading of a translation of Aesop’s fable *The North Wind and the Sun*. The 16 languages were distributed over four language families: (1) Romance (French, Italian, Spanish, and Catalan). (2) Germanic (German, English, Icelandic, and Danish). (3) Slavic (Russian, Polish, Serbo-Croatian, and Ukrainian). (4) Other smaller languages or isolates (Hungarian, Greek, Basque, and Welsh) – see also Figure 1 with a map of the languages.

### Phonetic Measures

***Speech rate*** equated to the number of syllables per second (see Coupé et al. (2019) for a discussion on how to calculate speech rate). A researcher with linguistic-phonetic training auditorily calculated speech rate for each language with the aid of a digital audio workstation (Adobe Audition [Computer software], 2018, Version 11.1.0) and visual control of the scripts. Silences longer than 50 milliseconds were excluded and only stretches with a continuous speech signal of at least one second were considered. Ten such speech streams were investigated for their syllable counts and the mean over those ten individual 1-s streams formed the final syllable rate per language. For quality control, speech rate for a second set of independent voice/language recordings of *The North Wind and the Sun* in the 16 European languages of the experiment was enumerated in the same way as described above and compared with the experimental set (set 1). The second set of recordings (henceforth called *the second set)*, was produced at a laboratory at the University of Vienna and consisted of all-female samples. There was a strong positive correlation between the experimental and the second set: *r* = 0.8, *p* = 0.000). For further quality control the speech rate data was compared to the values reported by an earlier publication (Coupé et al., 2019) resulting in a strong pos. correlation again (*r* = 0.8, *p* = 0.006) between our first experimental set and the sample published by Coupe et al., and another strong pos. correlation of *r* = 0.76 (*p* = 0.017) between our second set of recorded languages and the sample reported by Coupè.et al. (Note: the speech rate was not correlated with the speaker’s gender, *r* = 0.03; *p* = 0.8).

***The mean F0*** (fundamental frequency in hertz) of the voice recordings representing the 16 languages was extracted in Praat (Boersma and Weenink, 2017), a software package for speech analysis. Half of the recordings were spoken by male voices and half by female voices (*N* = 8). A continuous variable F0 was used to introduce a more neutral acoustic measure for the speaker’s gender. The F0 vector of the speech samples was also used to measure pitch modulations, melody, prosody.

#### F0-Trajectory Pitch Variation (Prosody) Measurement

The F0 pitch trajectories (pitch lists or vectors) were extracted by using Praat. Both sets of 16 voice recordings (first/main set of 16 languages, 50% female voices, and a second set of own voice recordings of the same 16 languages – all-female voices) were manually, visually and auditorily, checked and screened for pitch artifacts. An individual pitch range (see Table 1) was determined for every voice according to the artifact removal strategies in voice recordings as in Mayer (2019). Individual ranges were determined as cut-off frequencies and checked again for remaining artifacts that occurred due to hissing or creaky voices (high or low frequencies).

**TABLE 1:** Frequency ranges across languages in the first and the second set.

**Languages**	**The first (experiment) set**		**The second (control) set**
	**Female speakers**	**Male speakers**	**Female speakers**
Basque	125–300 Hz		175–350 Hz
Catalan		75–200 Hz	165–350 Hz
Croatian	120–260 Hz		145–350 Hz
Danish	115–280 Hz		145–350 Hz
English	115–300 Hz		170–350 Hz
French	130–250 Hz		140–350 Hz
German		80–230 Hz	135–350 Hz
Greek		80–200 Hz	155–400 Hz
Hungarian		75–300 Hz	135–270 Hz
Icelandic		75–200 Hz	165–350 Hz
Italian	120–370 Hz		120–290 Hz
Polish	90–400 Hz		155–330 Hz
Russian		75–200 Hz	140–350 Hz
Spanish	120–300 Hz		125–350 Hz
Ukrainian		75–250 Hz	155–400 Hz
Welsh	75–250 Hz		110–410 Hz

The extracted and artifact-controlled pitch trajectories were then converted into the international music cents scale with 55 Hz as reference frequency according to the formula [1,200 ^∗^ log2 (pitch vector in Hz/55)] to neutralize pitch effects between male and female speakers by transferring them into a normed reference frame (cents). From cents we converted further into semitones [1,200 cents or 12 semitones = 1 octave; 100 cents = 1 semitone]. Finally, we were interested in the pitch variation (melody of speech, voice modulations, and prosody) and calculated the variance or SD (standard deviation) of the music cents and semitones as ***Pitch variation***.

### Other Measures

***Lexical distance*** between languages (hereinafter the ***Serva-Petroni distance***) was estimated to control the distance between L1 of the perceivers (participants) and the 16 experimental languages. The ***Serva-Petroni distance*** based on a new automated method that uses the normalized Levenshtein distance developed by Serva and Petroni (2008), ranged from the minimal distance of 0 to the maximal distance of 1.

***Percentage learned as Lx*** refers to how much or how frequently a certain language of the experimental set (of those 16) was known or had previously been learned as a second/foreign language (Lx) by the participants. E.g. English has a score of 95% percent learned as Lx (foreign language) by our participants, whereas Greek, Basque, Catalan, Polish, Danish, Icelandic, and Ukrainian all got 0% because they were not learned by a single person of the sample as Lx.

Participants also self-reported on their ***musicality, singing ability***, and the number of and expertise in any musical instruments they play. Previous studies have shown that self-assessment serves as a reliable measure and is comparable to expert assessments (Christiner and Reiterer, 2013, 2015; Christiner et al., 2018).

### Procedure

We recruited primarily central European participants (*N* = 45). Following an online link, the participants listened to 16 European languages and evaluated them using opposite descriptors (e.g., beautiful vs ugly – see Table 2). The participants were instructed to use headphones. For the evaluations, an intuitive scale between 0 and 100 points with a slider was provided for each of the 22 adjective descriptors. We asked participants to estimate how familiar they were with the languages (self-perceived familiarity) and how much they liked the speakers’ voices. Furthermore, to control self-perceived familiarity, the participants were asked to name or guess the names of the languages they heard (if they were unsure, they could write whatever they associated with the language or name a language family). The later measure constituted ***Recognition rate***. A final comment box collected optional comments about the task and appeared after each language evaluation.

**TABLE 2:** Aesthetic descriptors.

**Scale**	**Negative descriptor**	**Positive descriptor**	**Source**
*Beauty*	Ugly	Beautiful	Giles and Niedzielski (1998)
*Coolness*	Uncool	Cool	Reiterer
*Culture*	Uneducated	Cultured	Giles and Niedzielski (1998)
*Elegance*	Inelegant	Elegant	Giles and Niedzielski (1998)
*Eroticism*	Unerotic	Erotic	Reiterer
*Fashion*	Unfashionable	Fashionable	Giles and Niedzielski (1998)
*Fun*	Boring	Fun	Giles and Niedzielski (1998)
*Generosity*	Stingy	Generous	Giles and Niedzielski (1998)
*Importance*	Marginal	Influential	Giles and Niedzielski (1998)
*Intelligence*	Stupid	Intelligent	Giles and Niedzielski (1998)
*Melody*	Tuneless	Melodic	Reiterer
*Memorability*	Unmemorable	Memorable	Reiterer
*Orderliness*	Chaotic	Orderly	Giles and Niedzielski (1998)
*Pleasantness*	Unpleasant	Pleasant	Giles and Niedzielski (1998)
*Romanticism*	Unromantic	Romantic	Reiterer
*Seductiveness*	Unseductive	Seductive	Reiterer
*Sexiness*	Unsexy	Sexy	Reiterer
*Softness*	Hard	Soft	Reiterer
*Status*	Low status	High status	Giles and Niedzielski (1998)
*Sweetness*	Harsh	Sweet	Reiterer
*Wealth*	Poor	Wealthy	Giles and Niedzielski (1998)
*Welcomingness*	Repellant	Welcoming	Giles and Niedzielski (1998)

All participants were informed about the main purpose of the research, its procedures, risks, and potential benefits. They volunteered to participate, signed a consent form, and received monetary compensation for their participation. The experiment was administered online using Gorilla Experiment Builder (Anwyl-Irvine et al., 2020), which generated a unique URL for each participant. All instructions and other texts featured in the experiment were presented in English. First, participants were asked to fill in a personal background questionnaire about their demographics and a language background questionnaire. Next, they were presented with a rating task featuring 17 recordings of languages in a randomized order (16 European languages of the study and 1 language doublet for control purposes). Participants were asked to rate each recording according to 22 aesthetic descriptors, available as sliding scales, and to provide further information about their impressions. They were required to use speakers or headphones and to complete the tasks using Mozilla Firefox as a default browser.

The instructions emphasized the importance of focusing on the sounds of the languages and not on the meaning of the presented text. Participants were told that there were no correct answers and were encouraged to use both extremes of the scale to their liking. They were allowed to listen to each recording as often as they wanted to. By the end of each trial, each language was evaluated in terms of its familiarity to a participant: the same sliding scale from 0 (“I don’t know this language”) to 100 (“I recognized this language”) was employed for this purpose. Participants could also guess which language they thought they have heard. Lastly, the speaker’s voice was evaluated following the same principle: 0 for “Very unpleasant voice” and 100 for “Very pleasant voice.” The optional subsection “Other impressions” allowed the participants to comment further on their experiences.

## Results

### Linguistic Variables and Aesthetic Ratings

As a part of the study, we measured linguistic background or control variables and the aesthetic ratings (see Table 3 for descriptive statistics).

**TABLE 3:** Descriptive statistics with the linguistic background variables and aesthetic ratings (0–100) with voice.

		**L1 speakers in mio**	**Ls learned as Lx in %**	**Recognition rate in %**	**Lexical distance**	**Overall mean score**	**BEAUTY**	**STATUS**	**EROS**	**SOFTNESS**	**ORDERLINESS**	**Melody**	**Voice**
N	Valid	16	16	16	14	16	16	16	16	16	16	16	16
	missing	0	0	0	2	0	0	0	0	0	0	0	0
Mean		38,1725	15,8638	46,6263	,770087	53,7174	56,6732	56,2097	45,0611	50,3681	54,4125	59,7556	59,8222
Median		30,5000	3,3100	34,4000	,790846	50,9086	53,7825	54,7986	41,2806	47,5611	52,6222	57,2333	59,6000
Standard Deviation SD		35,81612	25,06462	37,37447	,0854314	8,72251	8,29062	9,25013	12,12394	12,66038	9,14027	10,82261	9,42046
Range		109,69	95,00	95,60	,2602	29,90	25,94	30,35	39,86	49,69	32,33	35,76	34,24
Minimum		,31	,00	4,40	,6236	41,93	44,31	45,62	31,68	25,44	41,82	43,56	47,00
Maximum		110,00	95,00	100,00	,8838	71,83	70,24	75,97	71,54	75,13	74,16	79,31	81,24

*Here the 22 aesthetic descriptors are collapsed into five underlying factors (see the description of the principle component analysis applied below).*

All correlation coefficients of zero-order correlations are reported in Spearman’s Rho; we used the classical symbol “r” for these coefficients. Two-tailed testing was the default, and the results are reported at the significance level of *p* < 0.05, if not stated otherwise. For an overview correlation matrix of all variables see Figure 1).

**FIGURE 2:**
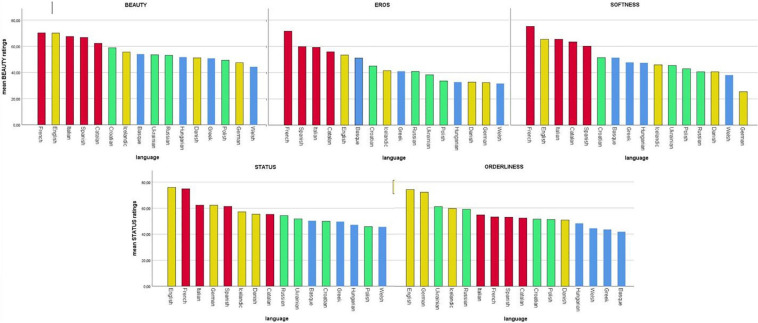
Aesthetic ratings, grouped by factors (Beauty, Eros, Softness, Status, and Orderliness), for all 16 languages, colored by language families (red = Romance, yellow = Germanic, green = Slavic, and blue = other).

**FIGURE 3:**
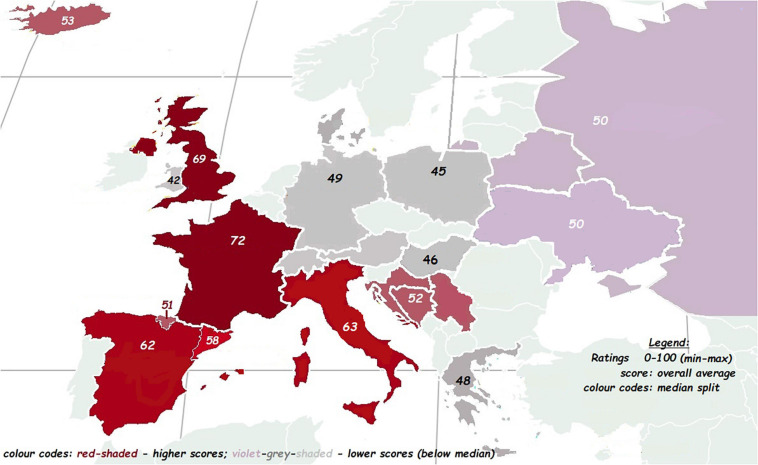
Mean (overall) aesthetic rating per language displayed on a geographical map. Color codes reflect the median split of the overall aesthetic ratings per language; red colors reflect higher ratings and violet or gray shaded reflect the lower ratings (0–100 min-max).

The factors ***Beauty, Eros, Softness, Status*, and *Orderliness*** were based on an exploratory factor analysis (EFA: described in Reiterer et al., 2020; see Table 4 below and Supplementary Figures 1S + 2S) which resulted in the reduction of 22 ratings to five factors based on *N* = 45. One of the original ratings was ***Melody*** (how melodious the language is). This adjective was subsumed under the factor ***Beauty*** by the EFA. However, since this adjective is of particular interest in studying the musical aspects of the ratings, we present the results of the melody ratings as well. Nevertheless, it should be noted that ***Melody*** is comprised as one of the eight scales under the factor ***Beauty***. ***Melody*** results that are reported below will always follow after the main five factors. The ratings of the five main factors are depicted in Figures 2, 3 where the ***Overall*** rating scores are color-coded on the geography map. For further details see Supplementary Tables 1S, 3S.

**TABLE 4:** The 22 aesthetic ratings collapsed into five factors (Beauty-yellow, Status-blue, Eros-red, Softness-orange, and Orderliness-green).

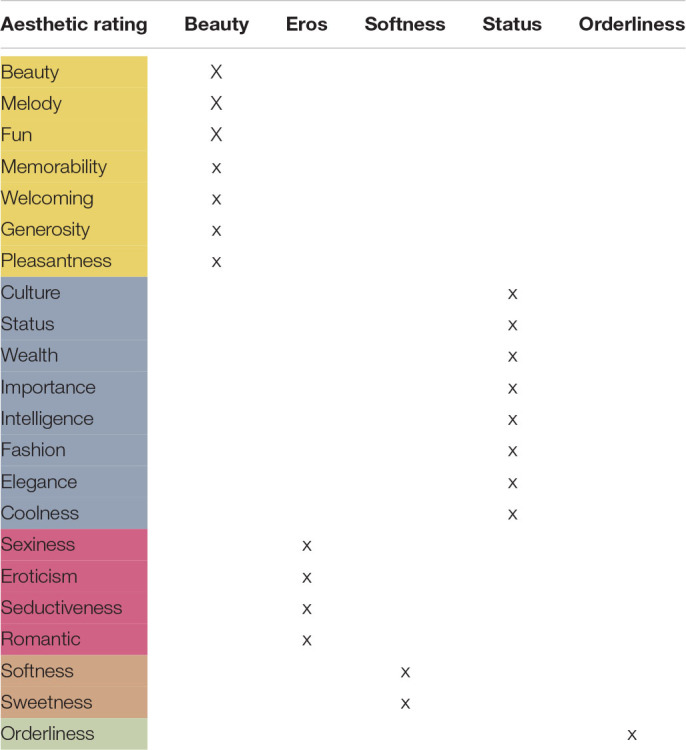

The languages with the highest ***Beauty*** ratings were French (70.24), English (70), and Italian (67.5). The lowest ***Beauty*** ratings were Welsh (44.3), German (47.4), and Polish (49.3). The languages with the highest ***Eros*** ratings were French (71.5), Spanish (59.7), and Italian (59.2) – a not surprising “Latin Lover effect.” The least erotic dubbed languages were Welsh (31.6), German (32.4), and Danish (32.7).

On the ***Softness/Sweetness*** scale, the highest languages were French (75), English (65.3) and Italian (65.3). The lowest scores were German (25.4), Welsh (38), and Danish (40.5).

On the ***Culture-Status*** scale, the highest languages were English (75.9), French (74.8), and Italian (62.3). The lowest scores were Welsh (45.6), Polish (45.8), and Hungarian (47). Note that, these ratings (unlike the first three) also correlate moderately and positively with ***L1 community size***.

The last factor found by EFA was ***Orderliness***: the highest languages were English (74), German (72), and Ukrainian (61). The lowest languages were Basque (41.8), Greek (43.5), and Welsh (44.4). Recall that we found a highly significant correlation between ***Orderliness***: and ***L1 community size*** with the more orderly languages having larger native speaker communities (German, English, and Ukrainian).

***Melody***, the single rating subsumed already under the factor ***Beauty***, yielded similar results to ***Eros***. The languages perceived as most melodious were French (79.3), Italian (76), and Spanish (72.2) – followed by English and Catalan. In the lowest range were German (43.5), Welsh (44.8), and Greek (51.6) – followed by Polish and Russian. Again, this picture resembles closely the ratings of the factors presented above. Perceived ***Melody*** ratings did not reflect the Hertz-based ***Melody*** measurements as measured by ***F0 pitch variation*** (see also ***Melody cents variance*** below).

***L1 community size*** (or a number of L1 speakers in millions) operationalized social-linguistic power relations. The smallest language community was Icelandic with only 31,000 speakers (0.31 mio) and the biggest community was Russian with 110 mio speakers. This variable was introduced to see the effects of cultural-political power on the aesthetic ratings. The results showed that there were no significant relationships between ***L1 community size*** and the ratings ***Eros***, ***Beauty*** and ***Sweetness*** [Spearmans’s *r* = 0.2, *p* = 0.5 (Eros); *r* = 0.2, *p* = 0.4 (Beauty), 0 = 0.1, *p* = 0.6 (Softness)]. However, L1 community size did affect the ratings ***Orderliness*** and marginally – ***Status*** [(*r* = 0.6, *p* = 0.01 (Orderliness), *r* = 0.5, *p* = 0.07 (Status)]. It is interesting to see that the greater ***L1 community size***, the higher the perception of a language status or “orderliness” – a clear sign of the impact of socio-political power on a perceived language status. However, this power does not transfer to the concepts that are more “emotional” or “aesthetic” in nature, such as language beauty, eroticity, and softness/sweetness. Further analysis revealed a strong and positive correlation with ***Recognition rate*** (*r* = 0.9, *p* = 0.000^∗∗^) and with ***Percentage learned as Lx*** (*r* = 0.7, *p* = 0.003^∗∗^). These results came as no surprise since they demonstrate the power effect, namely that the languages of the large communities (dominant languages) are familiar and recognized. These are also the languages that are typically acquired as foreign languages at mainstream educational institutions.

**FIGURE 4:**
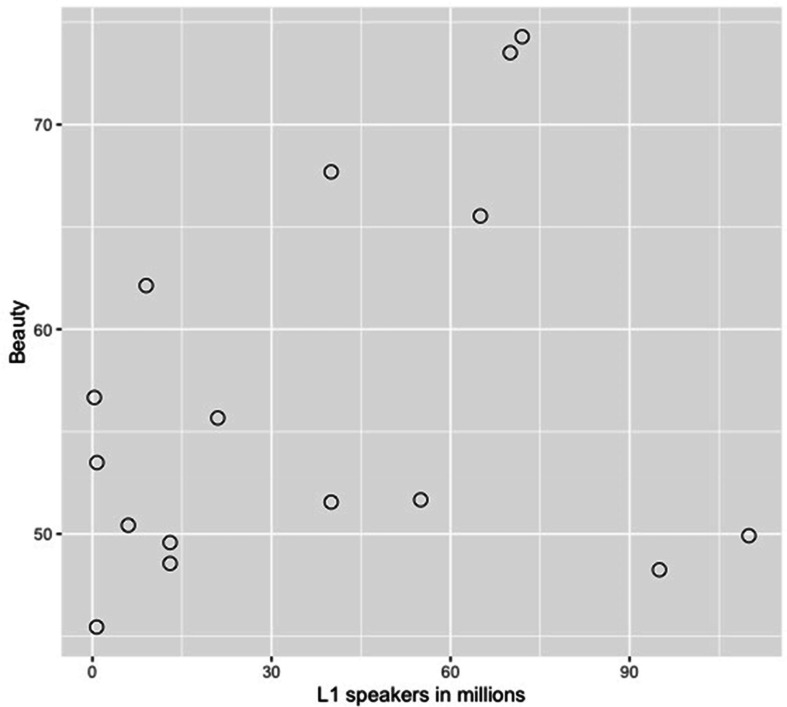
Scatterplot of the correlation between Beauty ratings and the number of speakers in Millions (community size).

Further, in Figure 1, ***Recognition rate*** is expressed as a percentage and ***L1 community size*** as a circle with a corresponding size. Figure 4 shows the (low) correlation between ***Beauty*** ratings and ***L1 community size***.

The next variable was ***Percentage learned as Lx*** or ***Percentage Lx***, the percentage of the participants that learned a given language as a foreign (L2, L3, and Lx) language. The share was rather low in total. Only 16% of all languages used in the experiment were learned as Lx by the participants. At the same time, the average number of foreign languages learned was three indicating a rather multilingual sample of participants. The experimental set consisted of many rarer languages that represented smaller language families, and that could be the reason why the overall share of Lx was rather small. Seven out of the 16 languages were not learned as a foreign language by anyone: Basque, Catalan, Danish, Greek, Icelandic, Polish, and Ukrainian. On the other hand, one language reached a 95% share of Lx learning and that was English, followed by German with 42% and French with 30%. Regression analysis showed that foreign language knowledge influenced ***Recognition rate***, and with that – the aesthetic ratings: ***Percentage Lx*** explained 40% variance in the aesthetic ratings (see a detailed discussion on familiarity and foreign language knowledge in Reiterer et al., 2020). Thus, in terms of language socio-political power, the **power of second language education** (foreign languages that are traditionally acquired in schools) is more influential than the size of the L1 community. We found that ***Percentage Lx*** correlated highly with ***Recognition rate*** (*r* = 0.9, *p* = 0.000^∗∗^), i.e., the more language is recognized, the more widely it has been learned as Lx. ***Percentage Lx*** also correlated positively with ***Status*** (*r* = 0.6, *p* = 0.01^∗^) and ***Orderliness*** (*r* = 0.5, *p* = 0.04^∗^): higher ***Status*** and ***Orderliness*** were ascribed to well-known languages. Concerning ***Beauty***, ***Eros***, and ***Softness***, there were non-significant relationships between these ratings and ***Percentage Lx***, as well as other acoustic variables.

**FIGURE 5:**
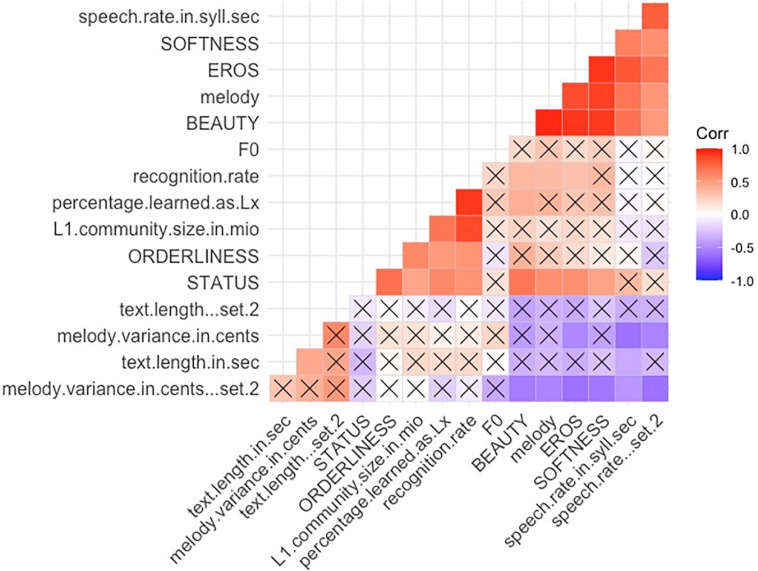
Zero Order (Spearman) Correlations Matrix Overview of all variables, *p* < 0.1 (*p* level was set to include trend-level correlations. For the chart displaying all correlation coefficients at *p* < 0.1^*trend*^, *p* < 0.05*, and *p* < 0.01** level see Supplementary Material Table S2).

***Recognition rate*** showed the percentage of languages that were identified correctly and reflected the familiarity with the languages. The most recognized languages were English, French, and German (all 100%). The least recognized languages were Basque (4%), Danish (9%), and Icelandic (9%). ***Recognition rate***, other than correlating to ***L1 community size*** and ***Percentage Lx***, correlated positively with ***Status*** and ***Orderliness*** (*r* = 0.55, *p* = 0.03^∗^; see also Figures 1, 5).

In the case of Basque, two-thirds of the participants when asked to identify the language commented alongside these lines: “some kind of Romance language either Portuguese, Romanian, or another one.” Thirteen participants could not provide any answer suggesting that it could be “an Indo-European language.” To check whether associating Basque with the Romance language had effects on aesthetic ratings, the independent samples t-test was performed (for the ratings of ***Beauty***, ***Eros***, ***Softness***, ***Status***, and **Orderliness**). The results showed no differences (*p* = 0.6 to.9) between the group that associated Basque with a Romance language and the group that did not.

The same picture emerged for the other two barely recognized languages. In the case of Danish 35 participants believed it to be either Dutch (the majority), German or “some kind of Germanic or Northern language.” Six participants could not identify it at all, with one participant suggesting it was Swahili. There was again no distinction in the group mean ratings as measured by the independent samples median test. In the case of Icelandic, a North Germanic language, the results were again similar with no significant group differences (t-tests) between those (*N* = 18) who thought it was “some kind of a Northern or Germanic or Scandinavian language” and those who (*N* = 23) though it was something else (e.g., a Finno-Ugric language with many participants suggesting Finnish and a few – a Romance or Slavic language). With all three unrecognized languages, we found no evidence of language family influence on the aesthetic ratings. This is particularly important for the case of Basque, where there is a danger to acoustically (and wrongly) classify it as a Romance language due to its century-long phonetic co-habitat with other Romance languages on the Iberian Peninsula.

One of the most diverse guesses was evoked by Welsh (the Celtic language family, Welsh was identified by 11% of the participants). Fourteen different languages and families were mentioned (other than “I don’t know”) in relation to Welsh, most pertaining to the Northern European regions, from the Germanic to Finno-Ugric language families, including guesses such as Lithuanian, Estonian, or Scandinavian. Some participants went as far as Arabic or Hebrew, with one particularly interesting answer: “at some point, it sounded like English with an accent…but it might be Bulgarian or Hungarian…not sure.” Many comments were linguistically interesting, such as “an English creole?” or “something like Gaelic – an Englishman who speaks Celtic.” For further details of the qualitative results (note, the guesses were obligatory, but the comments were optional) please see Supplementary Material.

The next variable was ***Lexical distance*** that referred to the distance between participants’ L1 and the languages of the study. This variable was used to control for typological influences of the mother tongue. ***Lexical distance*** was based on Levenshtein distances (Serva and Petroni, 2008; Petroni and Serva, 2010) and ranged from 0 (no distance between participant’s L1 and a given language) to 1 (maximal distance). The most distant language (to all participants) in the experimental set was Welsh with a coefficient of 0.88. The coefficients for Basque and Hungarian were not computed since no scores could be obtained for these language isolates (both languages do not belong to the Indo-European family). The closest languages for all participants on average were Croatian with a coefficient of 0.62 and German with 0.63. ***Lexical distance*** yielded no relationships (non-significant correlation coefficients, *N* = 14) to the aesthetic ratings (*r* = 0.04, *p* = 0.8 for ***Beauty***; *r* = 0.2, *p* = 0.5 for ***Eros***; *r* = 0.2, *p* = 0.5 for ***Softness***; *r* = -0.1, *p* = 0.7 for ***Status***, and *r* = -0.5, *p* = 0.07 for ***Orderliness***), reflecting no influence of L1 on the ratings. Only in the case of ***Orderliness***, there was a negative trend (yet non-significant): the more distant the language was, the less orderly it was perceived.

***Fundamental frequency (F0)*** was introduced to quantify acoustical differences between the languages voiced by male and female speakers. The male speakers (*N* = 8) had a F0 mean of 123 Hz, while female speakers had significantly higher values – 184 Hz on average. Such difference confirms previous research findings that describe gender-specific acoustic profiles. The overall mean of F0 in the sample (*N* = 16) was 153 Hz. The lowest F0 was for the Russian male voice and equated to 108 Hz, and the highest F0 was for the Italian and Polish female voices and both equated to 208 Hz. The mean F0 in the comparison recordings set (all female speakers) was 208 Hz (SD ± 24.7, range 165–243 Hz), with the highest pitched voice being the Ukrainian speaker and the lowest pitched voice – the Italian speaker. While ***F0*** correlated highly with ***Gender*** (the higher, the more female the voice of the recordings), *r* = 0.86, *p* = 0.000), the continuous variable **F0** in Hertz did not yield significant correlations with the likability ratings (*r* = 0.2 or below). However, a slightly different picture emerged when the calculations were carried out with the traditional binary male/female category.

Here, differences (the Mann Whitney U test for independent samples) between the genders emerged tendentially (*p* = 0.065) with the languages voiced by female speakers receiving higher likability ratings. The medians were: The ***Overall*** likeability score of 57 (female-voiced languages) vs 49 (male-voiced languages); ***Eros*** – 52 (female-voiced languages) vs 39 (male-voiced languages); ***Beauty*** – 63 (female-voiced languages) vs 54 (male-voiced languages); ***Softness*** – 56 (female-voiced languages) vs. 47 (male-voiced languages); ***Melody*** – 67 (female-voiced languages) vs. 55 (male-voiced languages); ***Status*** – 58 (female-voiced languages) vs 53 (male-voiced languages); ***Orderliness*** – 52 (female-voiced languages) vs 56 (male-voiced languages). Note that the differences for ***Status*** and ***Orderliness*** are not that discrepant. The gender effect is discussed extensively in Reiterer et al. (2020). While this might reflect a stereotyped well-known scenario, it is not clear why the continuous variable F0 – as a variable that belongs to a higher-order data level – does not confirm the same trend. As we know, gender can be predicted by F0 quite reliably.

### Acoustic-Phonetic Variables

Our variables of interest were acoustic-phonetic variables Table 5. Here, we included: ***Melody variance in cents*** (variance of F0 contour, so-called “melody of speech,” measured on the music cents scale), ***Melody variance/SD in semitones*** (same as above, just converted into a semitone scale), ***Speech rate*** (speech rate measured in syllables per second with both language sets), and ***Text length*** (the duration of the audio recording in seconds; see Table 3 for the summary).

**TABLE 5:** Descriptive statistics with the phonetic-acoustic variables for both language sets (*N* = 16).

		**F0 mean (set 1)**	**F0 mean (set 2)**	**Pitch contour in cents variance (set 1)**	**Pitch contour in semitones SD (set 1)**	**Pitch contour in cents variance (set 2)**	**Pitch contour in semitones SD (set 2)**	**Syllables / sec (set 1)**	**Syllables / sec (set 2)**	**syll / sec compared to (Coupé et al 2019)**	**Text recording in sec – (set 1)**	**Text recording in sec – (set 2)**
N	Valid	16	16	16	16	16	16	16	16	9	16	16
	missing	0	0	0	0	0	0	0	0	7	0	0
Mean		153,6804	208,1914	107855,5334	3,2006	87289,7104	2,8507	5,9063	5,8312	6,8691	37,3281	37,4931
Median		152,0234	212,3133	102388,8365	3,1867	67992,7190	2,6065	5,9000	5,7500	7,0650	39,0900	37,8700
Standard Deviation SD		34,40028	24,70695	50018,54414	,76029	55450,10240	,80150	,67845	,61937	,64028	4,42692	2,80527
Range		102,22	77,58	171114,42	2,61	221171,15	3,16	2,35	1,90	1,86	14,33	12,14
Minimum		107,88	165,71	38734,19	1,97	37026,79	1,92	4,85	4,90	5,87	29,73	33,06
Maximum		210,10	243,29	209848,60	4,58	258197,94	5,08	7,20	6,80	7,73	44,06	45,20

**FIGURE 6:**
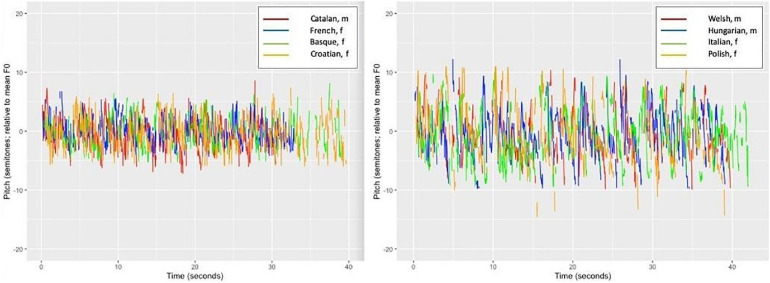
The pitch range (F0 fluctuations) for the four flattest (Catalan, French, Basque, and Croatian) and the four most variable/melodic (Welsh, Hungarian, Italian, and Polish) languages. F0 fluctuations are shown in terms of ±standard deviations of musical semitones.

**FIGURE 7:**
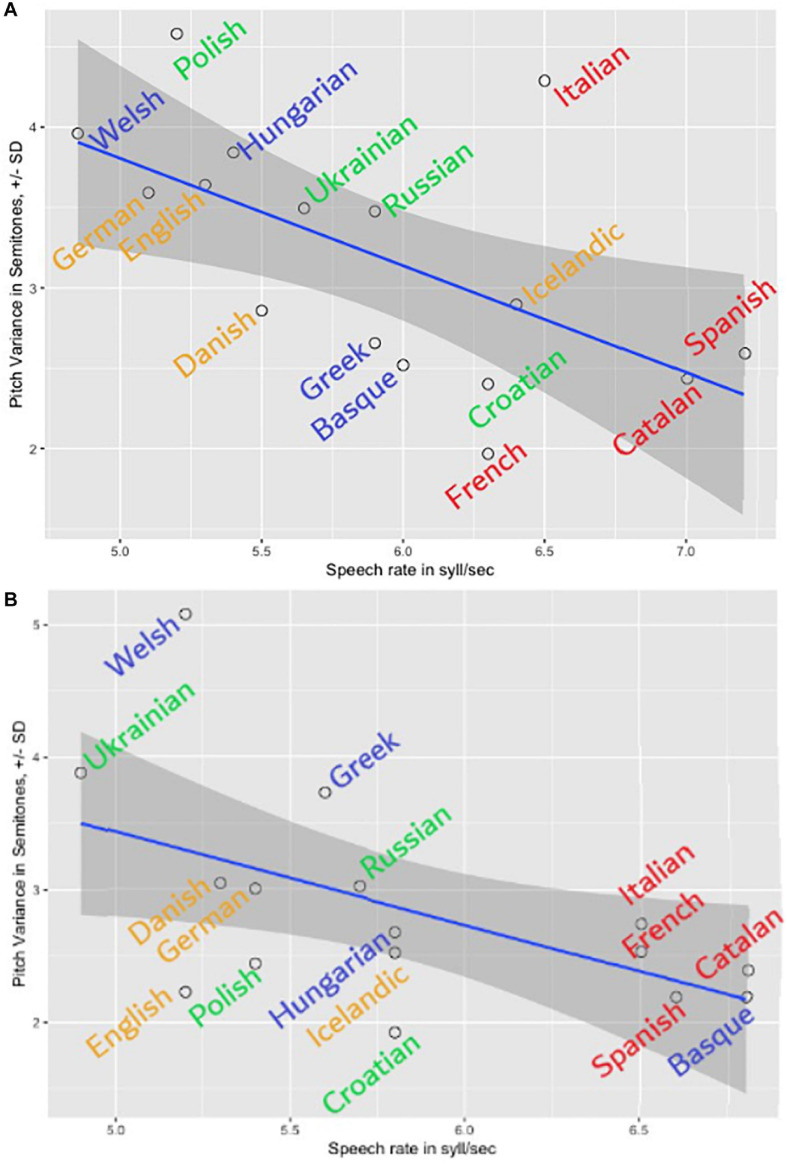
The speed-melody trade-off in the first **(A)** and the second **(B)** sets of language recordings: the slower the speech, the more pitch modulations are found.

***Melody ñents variance*** referred to the F0 fluctuations or trajectories that were translated into music cents and later into the semitone scale. To track speech melody in terms of musical melody (as opposed to the linguistic melody), we measured the original F0 speech contours on the basis of Hertz of the fundamental frequency in both language sets. The mean speech melody fluctuations expressed in standard deviations of semitones were ±3.2 semitones (SD of ±0.76) in the first set and ±2.9 semitones (SD of ±0.8) in the second set. The largest deviations (high variance, more F0 modulations) were ±4.6 or ±5 semitones, whereas the lowest fluctuations were ±2 or ±1.9 semitones, respectively, on average. The larger modulation ranges, such as ±5 semitones (considering ±) this means a range or tone interval of 9.2–10 semitones, which corresponds to almost one “sixth” of the octave range. This is a rather large range for speech that was produced by reading aloud neutral texts (containing no expressions of extreme emotions or cries). When compared to a song, one could realize a restricted variety of songs with a pitch range of one sixth. The other extreme (4 semitones = more or less equivalent to a “second” pitch interval in music) is suboptimal for realizing a tune or a song melody. Thus, we could not think of any tune or song which suffices only on a pitch range of one “second” (only the very beginning of the Beatles song “*Yesterday*” could be started on that interval). The least melodious languages in our set were comparable to such a “song.” The least melody/pitch variance [i.e., flattest melody) was found for French (2), Croatian (2.4), and Catalan (2.4), followed by Basque (2.5). In the second language set, similarly, these languages were Croatian (1.9), Basque (2.2), and Spanish (2.2). The languages with the highest pitch modulations in the first set were Polish (4.6), Italian (4.3), and Welsh (4), followed by Hungarian (3.8). In the second set, we had Welsh (5), Ukrainian (3.9), and Greek (3.7) with this range (see also Figures 6, 7). With the pitch intervals found for Italian, Polish, and Welsh (a fourth, fifth, and a sixth pitch interval in terms of F0 variance), one could try and sing children’s songs such as *All my little ducklings* or *Twinkle, twinkle little star. Even* these rather wide speech pitch ranges are not comparable to the much more variant pitch ranges/intervals used in songs, typically performed in most musical styles. Thus, to summarize, we found that music uses far wider pitch ranges than language expressed by speech melody. Speech is rather flat when compared to music.

**FIGURE 8:**
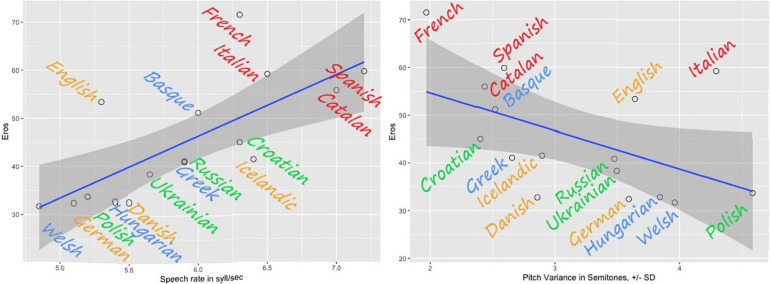
The relationships between the Eros factor and the speech rate (on the **left**) and the Eros factor and the pitch variance (on the **right**). The highest Eros ratings are assigned to faster flatter languages.

***Melody cents variance*** showed a series of significant correlations. First and foremost, it correlated negatively with ***Speech rate*** (*r* = −0.6, *p* < 0.01^∗∗^), meaning higher ***Melody ñents variance*** went hand in hand with lower numbers of syllables per second (see also Figures 7A,B). The same result (*r* = −0.6, *p* < 0.01^∗∗^) – the speed-melody trade-off – we observed in the totally independent second set of language recordings (Figure 7B). Thus, the slower the speech was, the more pitch modulations were found; the opposite was also true – the faster the speech, the fewer (F0) modulations (flatter, fewer semitones up and down) were present in the recording. In other words, the speakers who “sang” (modulated more in terms of pitch) slowed down; and the speakers who kept their F0 stable spoke faster. Interestingly, a significant negative correlation was found between ***Melody ñents variance*** and ***Eros*** (*r* = -0.5; *p* = 0.04). Thus, higher ***Eros*** ratings were in line with **lower** and not higher **speech melody**, as it would be suspected. It was the faster speech and not the melody that participants found erotic (see Figure 8). There were no significant correlations (below or around 0.2) between F0 distribution as a marker of an acoustic ***Gender*** and ***Melody cents variance*** in the first (gender-mixed) language set. This finding did not confirm the common belief that female speakers display more speech melody or prosody. However, larger recording sets are needed to investigate the gender aspect thoroughly.

Furthermore, in terms of ***Melody ñents variance*** the two language sets showed a trend toward a weak or moderate positive correlation, but this was not significant (*r* = 0.4, *p* = 0.12). This indicates that pitch ranges and speech melodies are subject to both, language inherent features and individual differences in voice, and more research is necessary to show how stable the prosody characteristics are across languages (inherent to languages). We discuss this question further in *Limitations*.

***Speech rate*** in syllable per second was measured as another acoustic-phonetic feature of speech. ***Speech rate*** was compared to the second set of recordings matched for language and text type (*The Northwind and the Sun*) to check the reproducibility and reliability. The sets correlated highly and positively (*r* = 0.8, *p* = 0.000^∗∗^). The paired samples t-test showed no significant difference between both language sets: the mean for the first set was 5.9 syllables per second (±0.6) and the mean for the second set was 5.8 syllables per second (±0.6). Next, we compared our ***Speech rate*** to the same variable in the study conducted by Coupé et al. (2019). In their study, ***Speech rate*** was measured across a variety of world languages and based on numerous text genres and speakers. We had nine overlapping languages with their dataset: Basque, Catalan, Serbo-Croatian, English, French, German, Hungarian, Italian, and Spanish. We found that both our language sets (first and second) correlated highly with ***Speech rate*** Coupè and colleagues reported (the first set: ***r* = 0.72; *p* = 0.03^∗^**; the second set: ***r* = 0.68, *p* = 0.04^∗^**).

The languages with the highest ***Speech rate*** in the first set were Spanish (7.2 syll/sec), Catalan (7 syll/sec), and Italian (6.5 syll/sec). The lowest ***Speech rate*** were found for Welsh (4.8 syll/sec), German (5.1 syll/sec), and Polish (5.2 syll/sec). Furthermore, ***Speech rate*** yielded strong positive correlations to all the aesthetic ratings, except for ***Status*** and ***Orderliness***: with ***Eros*** (*r* = 0.8, *p* = 0.000^∗∗^), with ***Beauty*** (*r* = 0.7, *p* = 0.002^∗∗^), with ***Melody*** *r* = 0.7, *p* = 003^∗∗^), and with ***Softness*** *r* = 0.6, *p* = 0.007^∗∗^) – the faster, the better. These perceptions unanimously show that the faster the spoken speech, the higher the likability ratings. The example of this result is illustrated in Figures 8A,B with ***Eros*** ratings. Two “big” languages form outliers to the correlation: English and French. French reaches a rather high ***Eros*** rating in proportion to its speed, and so does English. However, since both languages are amongst the most popular foreign languages learned and institutionalized this finding should not be that surprising.

Since it is still unclear how pitch ranges and F0 variance fluctuate across languages (and how much they vary across individuals as personal voice/prosody traits), we think that the pitch variations found here reflect both: language-inherent prosodic characteristics and voice-individual characteristics (see more notes on voice in the Discussion).

***Text length*** (refers to the length of the recordings in seconds) was used to control for the influences of the inter-individual speaker or language-related (lexicon, translation, word-formation) differences of the text. Although the storyline of *The Northwind and the Sun* is the same across languages, every language uses its own morpho-syntactic and lexico-semantic rules to realize the text, and moreover many different versions of the translations exist. Notwithstanding, despite all the idiosyncrasy, we found no significant correlations with all the variables, but two: in the first set and the second set of independently collected voice recordings’ text length showed a marginally significant moderate correlation (*r* = 0.46; p.076) and there is a significant positive high correlation between ***Melody cents variance* and *Text length*** (*r* = 0.6, *p* = 0.01^∗^) in the second set and there was a similar one at trend level in the first set of recordings (*r* = 0.44, *p* = 0.085). The positive correlation means that the more speech melody is modulated along the pitch trajectory, the longer it takes to voice the text. Lower ***Melody cents variance*** or flatter voices read the text quicker. This hints toward an articulatory trade-off between time and vocal space (e.g., to “sing” a sentence takes longer).

### Further Correlations: Musicality and Singing Ability

We asked participants (*N* = 45) to self-report on their musicality (the scale of 0–10), the number of instruments played, and singing ability. As a result, ***Singing ability*** correlated with the likability ratings, but also with ***Recognition rate***, i.e., the familiarity with the languages (in fact, all music-related scores correlated significantly with the recognition rate: Pearson’s r ranged between 0.38^∗∗^ and 0.47^∗∗^). The higher the music expertise/practice, the higher also the foreign language expertise. Musical people were more successful in guessing the languages they heard. Even though it did not necessarily imply that they spoke these languages, it seemed that they had a better feeling or “ear” for the languages overall. There was a trend between ***Singing ability*** and the number of foreign languages spoken (*r* = 0.3, *p* = 0.052). ***Singing ability*** also correlated positively with ***Beauty*** (*r* = 0.3, *p* = 0.04), and ***Eros*** (*r* = 0.3, *p* = 0.02), and not significantly – with ***Softness***, ***Melody***, ***Status***, ***Orderliness***, or ***Voice*** ratings. ***Musicality*** correlated highly (*r* = 0.75^∗∗^, *p* = 0.000) with ***Singing ability***, but also with ***Beauty*** (*r* = 0.3, *p* = 0.02) and ***Status*** (*r* = 0.3, *p* = 0.04) and not with the other factors (***Melody***, ***Orderliness***, ***Softness***, ***Voice***, and ***Eros***). In sum, a sensitivity toward music and singing was connected to the enhanced perception of language beauty, eroticity, and status. Musical people were also better language guessers: it was enough for some of them to get exposure to the sound shape of the language sample once to identify the language correctly. It could be that having a good ear for music works for language as well.

### Voice as a Nuisance Co-variate

***Voice*** judgments correlated highly with the following aesthetic ratings: ***Beauty***, ***Eros***, ***Softness***, and ***Melody*** (*r* > 0.8, *p* = 0.000^∗∗^) and ***Status*** (*r* = 0.5, *p* = 0.04), but not with ***Orderliness*** (*r* = 0.2, *p* = 0.36). Furthermore, ***Voice*** correlated significantly with ***Speech rate*** (*r* = 0.6, *p* = 0.01). See Discussion for the relevant comments on this trend.

## Discussion

It is widely believed that some languages sound more musical than others, and attempts to identify the music-in-the-language have been around for at least several centuries. Charles V (1500 –1558), Holy Roman Emperor, dryly remarked, “I speak Spanish to God, Italian to women, French to men, and German to my horse” (Brunner, 2014). Prompted by comments such as this, the present article aims to capture the quantitative sources behind the aesthetic judgments of cross-linguistic stimuli and explains the music-like effect that some languages, particularly the Romance languages, convey to listeners.

### Familiarity

Participants in this study were familiar with many of the languages we used: 100% recognized German, French, and English, 93% recognized Italian, and 78% recognized Spanish. The less recognized languages were Icelandic, Danish, and Basque, with a recognition rate of 9, 9, and 4%, respectively. Welsh evoked the most guesses.

Overall, participants derived more pleasure from listening to the languages they recognized (except German; on the contrary, Basque or Icelandic were highly rated but barely recognized) because familiar languages seem more beautiful, erotic and of a higher cultural status. Decades of research into the connection between familiarity and liking have shown a robust positive correlation between the two (e.g., Birch and Marlin, 1982), the gist of which is summarized by an oft-quoted German proverb: “What a farmer does not know, he does not eat.” Evolutionary psychologists explain this preference by saying that familiar objects are perceived as favorable because they have been proven harmless after the initial exposure (Bornstein, 1989). More recent socio-cognitive explanations point to a facilitation effect, which occurs when a stimulus is processed on a second or third-occasion (Winkielman and Cacioppo, 2001; Reber et al., 2004). Thus, familiar languages, requiring less effort to process, might be perceived as sounding more pleasant because they are easily recognized. In this sense, the auditory pleasure derived from listening to the sounds of language resembles the pleasure derived from music: anticipation or expectancy (previous knowledge of language or music) is the essential mechanism for a pleasurable experience (Steinbeis et al., 2006; Vuust and Kringelbach, 2010).

Nevertheless, our participants did not prefer languages that were their native languages (L1s) or close to L1s, the same linguistic family they usually spoke. Rather, they were influenced by the familiarity associated with a foreign or second language-learning experience (the so-called “exotic touch”); the more languages they spoke, the more they enjoyed the sound of foreign languages. In this regard, the Serva-Petroni lexical distance (Serva and Petroni, 2008) mattered: distant but familiar languages were more welcome than less distant but familiar languages. This balance between sounding exotic and being familiar influenced (i.e., significantly correlated with) most of the aesthetic ratings, namely, culture-status, sweet-softness, and eroticity, which again echoed the findings of similar research in music (Eisentraut, 2012).

Despite the positive effect of facility by familiarity, several languages did not conform to this pattern: e.g., German was recognized by 100% of participants but received unfavorable aesthetic ratings (only in orderliness and culture-status), whereas the unrecognizable Basque and Icelandic languages enjoyed favorable ratings. On the other hand, exotic, and barely recognizable Basque came sixth in eroticity rating and seventh in sweetness and softness. Thus, with Basque and Icelandic, it appears to be that the language’s sound shape and not its socio-cultural associations evokes pleasurable responses.

Certainly, when dealing with natural (and not artificial) languages and aesthetic judgments, it is almost impossible to separate the socio-cultural influences (e.g., enjoying the sounds of Italian because Italy is beautiful) from the language’s acoustic contributors (e.g., Italian is spoken rapidly with a high sonority index). In our previous study (Reiterer et al., 2020), we concluded that it is the complex interplay of socio-cultural factors, idiosyncratic properties of voice, and language-specific acoustic features that account for aesthetic judgments about language. In the present study, the goal was to focus on language-specific features exclusively. Nevertheless, we looked at one socio-cultural aspect – the size of the L1 speaker’s community and observed a connection between it and the higher ratings for orderliness and culture-status. While we might plausibly assume that “big” languages are empowered ecologically, as they have greater prestige in comparison to minority languages, we might also expect participants to rate such languages (e.g., German and Russian) as highly ordered with a high culture status. However, we found no relationships between community size and beauty, eroticity, or melody ratings. It seems that our participants’ linguistic aesthetic judgments were unaffected by this soci-cultural factor, i.e., they did not find high-status languages to sound more pleasant.

### Musical Abilities and Individual Differences

Not all participants were affected by the acoustic features of the language recordings to the same degree. In our study, participants with higher self-perceived musical abilities (particularly in singing) tended to find languages more beautiful and erotic and assigned a higher social status to them. Such individual differences might be explained by neural mechanisms that underlie language and music. Taking congenital amusia (a neurodevelopmental disorder commonly known as “tone-deafness”) as one extreme example and an exceptional musical talent as another, individuals vary in the ways they process musical stimuli. Previous research has shown that emotional prosody in speech, when other linguistic information is absent, is processed similarly to music (Patel et al., 2005; Hutchins et al., 2010; Zhishuai et al., 2017), so that a non-musical person might be unaffected by the emotional valence of the acoustic features that language conveys. However, the opposite might also be true: sensitivity to emotional speech prosody or sound properties of language might be enhanced in individuals who can process and interpret music well (Thompson et al., 2012).

Investigating a massed repetition effect inducing a perceptual transformation from speech to a song, Falk et al. (2014) found individual differences in sensitivity to underlying acoustic cues. Thus, rhythmically sensitive participants experienced the speech-to-music transformation more often compared to other less sensitive participants. In their study, a surprising finding was that professional musicians are not always high-perceivers, perhaps because they might have higher criteria for auditory signals to be interpreted as musical. On the other hand, amateurs tend to evaluate speech as music much more readily. In this regard, musicality that is connected to “phonetic chill,” the intense pleasure one experiences when listening to the sounds of language, should be interpreted not only in terms of professional training but also as a stand-alone individual perceptual and productive ability and strategy (Christiner and Reiterer, 2015). Assaneo et al. (2019) similarly observed that formal musical training does not explain an enhanced ability to synchronize motor output to auditory input (e.g., tapping to music). Synchronization type is a consistent individual trait supported by functional and/or structural brain differences and influences, among other tasks, language learning outcomes. Assaneo et al. (2019) found that high synchronizers were also better language learners, an association confirmed by the present study in which participants with musical abilities spoke, on average, more foreign languages than their non- or less-musical peers. The connection between musicality, especially singing, and foreign language aptitude appears in children as early as kindergarten, as several studies show (e.g., Nardo and Reiterer, 2009; Christiner and Reiterer, 2018; Christiner et al., 2018).

### The Heartbeat of Language Is Rhythm

While some languages are spoken faster than others, yet, the rate of information they convey is much the same. Coupé et al. (2019) analyzed 17 languages from nine language families and reported comparable average rates at which information is emitted across languages (the channel capacity). In their sample, the fastest languages in the sample were Japanese, Spanish and Basque; the slowest were Yeu Chinese/Cantonese, Vietnamese and Thai. Because speech rate does not influence encoding efficiency, it might nevertheless have aesthetic value. Our participants found faster languages (Spanish, Catalan, Italian, and French) more beautiful, erotic and ‘melodious’ but less orderly and more chaotic. Participants characterized Spanish as sounding “strong,” “convincing,” and “decisive.” Catalan sounded “too fast.” For Italian, one participant said: “pleasant flow” and for French – “wunderschön gesprochen” and “in this case I experienced slight ASMR” (autonomous sensory meridian response, i.e. chill). English was also one of the slowest languages in our sample, yet, it was difficult for participants to evaluate this language objectively due to a high proficiency level: “Initially I couldn’t help not to judge the language including the meaning. The experience of the sound immediately gave me visuals and the atmosphere.” Some of our participants labeled slower languages as sounding “harsh, hard to pronounce” (German), “middle age/older” (Polish/Danish), and “the pronunciation seems very hard” (Welsh).

Previous research that analyzed how speech rate and tempo in music relate to emotions demonstrated that increased speed in both domains is associated with high-arousal or “active” emotions (Juslin and Laukka, 2003; Ma and Thompson, 2015), such as happiness, the anger and fear. While we are unaware of research connecting speech rate with aesthetic judgments, one way to interpret our findings is to look at the connection between the rate of speech and syllabic structure. The most likable languages (in our study) employ primarily the CV structure: e.g., 58% of syllables in Italian are CV syllables, compared to 31% in German (Rabanus, 2003). Such structures imply not only a greater articulatory economy (Janson, 1986) but also produce a simple rhythmic pattern with regular syllabic pulses that might boost the speech rate. Slower languages (e.g., German and Polish) have more elaborate syllabic structures that might be perceived as syncopated rhythms – complex and ambiguous rhythms that sound “off-beat.” Such rhythms stress weak positions in the metrical structure while leaving nearby strong positions “empty,” or without stress. Fitch and Rosenfeld (2007) investigated syncopation in music and concluded that its rhythms are difficult to process and remember since they require listeners to reset their internal pulse representations. In the case of languages, syncopated rhythms might also call for greater cognitive and articulatory effort, leading to slower speech rate, and negative aesthetic ratings (However, since syncopation in language is rarely explored, this must be said with caution).

Dellwo and Wagner (2003) found that the consonantal interval measure correlates negatively with speech rate, meaning that languages with complex consonant clusters (e.g., German, Polish, or Russian) would have a slower speech rate compared to languages that lack this feature (e.g., Italian, French, and Spanish). They also found a positive correlation between speech rate and sonority that is tightly connected to vocalic share – vowels are the most sonorous sounds in most languages. One would expect that languages with the predominant CV structure normally have more vowels, and therefore, a higher sonority index. In our previous study (Reiterer et al., 2020) we found a positive correlation between sonority and beauty ratings: more sonorous languages were also perceived as sounding more beautiful and erotic.

The CV syllabic structure is faster to perceive, process and produce, and when the structure is repeated it might produce a music-like effect. Falk et al. (2014) looked at the repetition effects that induce a perceptual transformation from speech to song – the situation, in which a spoken sentence repeated several times begins to resemble a song. They explained the emergence of musical percepts by a more detailed acoustic encoding that is facilitated as a result of repetition. In other words, when the same verbal structure is repeated its content fails to become of primary importance (in our study, most languages were not understood semantically) and the acoustic characteristics (e.g., melody, rhythm) that did not matter before become more salient. Even though this study was conducted with utterances and not syllables, we can assume that a similar perceptual strategy might be applied to a repeated syllable. If a syllabic structure is the same or similar every time (e.g., “banana”), cognitive resources, freed from attending to a varying syllabic structure and associating with its rhythmic (syncopated) complexity, are used for the enhanced perception of the acoustic properties creating a music-like effect.

Finally, a recent preliminary investigation by Lin and Rathcke (2020) into the anchor of sensorimotor synchronization suggests that the moments of local maximal energy increase (maxD) as well as vowel onsets, are prominent acoustic landmarks for rhythmic attention and synchronization with speech. Based on these findings, languages with more vowels could be perceived as possessing a faster rhythm (in our sample, Basque and French had the highest vocalic share, German and Polish the lowest; Reiterer et al., 2020). Infant et al. (2013) measured rhythm perception based on the amplitude modulation structure of the speech envelope, which is also connected to syllabic nuclei and vowels, and observed that syllable-timed languages (e.g., Spanish or French) indeed have faster rates than stress-timed languages (e.g., German or Russian).

### The Melody Paradox

When we speak of melody, we traditionally mean music and music-like stimuli (environmental sounds, the melody of the voice, etc.). However, in linguistics melody refers to an organized pitch pattern in speech (Patel, 2010). In the present study, we employ ‘speech melody’ in its musical sense – how melodiously a language sounds, the music-in-the-language. Our results showed that languages with fewer pitch variations (F0 contour as measured in hertz/cents/semitones) are perceived as more erotic and melodious. Except for Italian, the Romance languages (Catalan, Spanish and French leading the list) show the flattest intonation contour. At the opposite end were Welsh, English, and Polish (and Italian), which used an impressive range of pitch variation. Compared to music semitones, Polish would sound like *My bonnie lies over the ocean/*/*Nobody knows the trouble I’ve seen*, and, Catalan, the most atonal, would sound like the first bar of two bars in *Strangers in the night* or *Yesterday*. This surprising finding can be summarized thus: the most “melodious” languages are those without melody!

It has been observed previously that linguistic intonation indeed lacks the complexity and aesthetic potency of musical pitch, which has an elaborate system of intervals with a rich network of pitch relations. Patel (2010) commented that: “Intonation contours are aesthetically inert, as evidenced by the fact that people rarely hum intonation contours or find themselves captivated by the pitch patterns of speech” (p. 184). Indeed, pitch has a different function in speech. In music, it is an aesthetic object, but in speech, pitch has a practical purpose – to convey structural information. Since pitch is not the only linguistic feature responsible for this function, spoken language, compared to music, is rather atonal (Chow and Brown, 2018), making it very hard to derive an aesthetic pleasure from linguistic intonation. Even when pitch varies minimally it does not affect comprehension significantly.

A recent study by Albouy et al. (2020) demonstrated that perception of speech is most affected by the degradation of information in the temporal dimension (rhythm), whereas perception of music is most affected by degradation in the spectral dimension (pitch). The authors propose that these two domains employ opposite extremes of the spectro-temporal continuum, which is reflected neurally as two specialized complementary systems, one in each hemisphere. In the light of these new findings, a wide range of F0 variation throughout an utterance is not essential for comprehension. The temporal as well as timbral (Reiterer et al., 2008) information, on the other hand, is a more crucial dimension for speech. In this regard, flat and fast Romance languages might be processed by naive listeners with greater ease and generate positive judgments for that reason. This means that the ‘melodiousness’ of French and Spanish is rooted in the rhythm and socio-cultural stereotypes attached to these languages and not in the actual speech melody, i.e., pitch variation.

In our study, the tradeoff between pitch and rate is surprisingly consistent across languages: the more pitch is modulated, the slower the speech rate. The opposite is also true: the less pitch is modulated, the faster the speech rate. The idea that auditory cognition depends on the processing of spectro-temporal energy patterns and that these features often trade-off against one another is not new and has been demonstrated before (e.g., Elliott and Theunissen, 2009; Flinker et al., 2019). Speech can be understood with either very coarse spectral information or very coarse temporal information (Arai and Greenberg, 1998). In the present study, the languages display various ratios between pitch variation and rate, with the Romance languages being the flattest and the fastest, a combination that can hardly be said to be melodious unless the claimant is a fan of hip-hop. Interestingly, that Spanish has been often called a “machine gun” language because of its fast and flat sound. On the other hand, the Germanic and Slavic language families show greater variety in terms of pitch, and therefore, are more melodious in the linguistic sense of the term. At the same time, these languages are slower, thus, producing an opera-like effect – the prolonged intervals of “singing” that take time and allow for more pitch variations.

Just as languages can be classified loosely into tempo-dominant and pitch-dominant, listeners might be sensitive to one domain more than the other. In a recent study, Christiner et al. (2018) found that listeners with superior rhythmic discrimination ability as measured by a subtest of IMMA (a test of musicality; Gordon, 1982) imitated an unfamiliar language, Tagalog, better than they did unfamiliar Chinese. Because Tagalog is a non-tonal language, therefore, its rhythmical organization can be predominantly recognized by naïve listeners. In marked contrast, a singing ability appeared to be key to imitating Chinese, a tonal language where pitch plays an important meaning-bearing function. The authors concluded that people vary in the type of specific acoustic features they rely on when processing utterances in an unfamiliar language. These individual differences do not have to be structured by a native language alone; they can be determined by the type of musical training – e.g., the type of instrument played (Schneider et al., 2002) – or innate abilities; e.g., singing (Oikkonen et al., 2015). To shed light on the nature of aesthetic preferences and to explain phonetic aptitude in some language learners, we recommend that the interplay between language typology and individual differences in auditory processing should be researched further (Kogan and Mora, 2017).

### The Case of Italian

Italian was the only language that did not demonstrate a clear trade-off between speech rate and intonation (at least in the first set of recordings): both fast and varied in pitch, it remains a linguistic enigma. More research – and certainly more recordings coming from a variety of speakers – is needed to understand how Italian speakers (as it was one the outlier cases here) manage to be fast and varied in pitch at the same time. However, as the second set of independent recordings showed, Italian was a perfect fit to the trade-off between speed and melody.

Another interpretation underlying this trade-off phenomenon in general could be that it reflects a more basic physiological mechanism, namely a sort of “Heisenbergian” trade-off between space and time resolution. It is imaginable that our vocal articulators are not optimized for producing melodious (pitch-intense) but precise and highly intelligible speech, e.g. by spanning octaves (like in arias), and being very fast at the same time (like in casual conversation). Articulatory movements (larynx/pharynx, lungs, and mouth cavity) have to be coordinated in space and time. Usually, song is slower than speech, for if we sang everything we wanted to say, we would need a multiple of time for information exchange.

### Limitations

One of this study’s principal limitations is that the languages it used are represented by single speakers (one speaker per language). This methodological decision was dictated by the study’s preliminary nature: to build a larger repository of language recordings (in terms of speakers per language and the number of languages) at a later stage. Although this is not an ideal scenario, previous studies indicated that while there is wide interspeaker variation in an acoustic parameter within a language, this variation is also structured by language (Coupé et al., 2019). In other words, individual speech behavior is not due to individual characteristics alone but is further defined and guided by the language being spoken. Yet, it would be optimal to have several speakers, preferably of the same gender, voicing the same language. In fact, the second set of language recordings was collected after the present study was conducted to confirm some of the acoustic-phonetic phenomena observed in the first set. The *speed-melody tradeoff* was, in fact, exactly replicated in the second set, as well as the ordering of languages in terms of the *speech rate*. However, in terms of the pitch variance (melody), the two sets only showed a weak positive trend indicating that the pitch-related results should be considered as reflecting both, individual (speaker inherent) and more global linguistic-typological (language inherent) traits.

In regard to gender, in our previous study (Reiterer et al., 2020) we found that languages presented by female voices were rated significantly higher on average than those presented by male voices. This finding confirms previous research that addressed this methodological issue (McMinn et al., 1993; Wilding and Cook, 2000; Whipple and McManamon, 2002; Edworthy et al., 2003). In this study we used a continuous variable for F0 rather than a binary male/female variable, and the previously observed connection between the speaker’s gender and the aesthetic ratings was not present this time. Also, we did not find a connection between female speakers and the average pitch variance or speech rate.

As well as inter-speaker voice differences, there are also individual differences in how a voice is processed by listeners. Some listeners have an enhanced ability to extract, evaluate, and categorize non-linguistic information available in voices (Brück et al., 2011). Such voice-sensitive individuals might experience any spoken stimuli more intensely, and therefore, might be more susceptible to phonetic chill. Studies employing brain imaging techniques would be particularly helpful to account for this variable. The voice as a nuisance variable should be addressed in more detail in future studies.

Finally, the question of familiarity with some of the languages in the study is an important methodological decision that one has to make. We carefully measured the familiarity aspect not only by asking participants to guess the language they were listening to but also by including additional questions about participants’ second language education and languages spoken (at which proficiency levels) besides their mother tongue. This information was included in the analysis (polyglot factor) and reported accordingly (Reiterer et al., 2020). By no means do we deny the influence of familiarity on participant’s judgments – on the contrary, we would like to endorse that it is a complex interplay between the familiarity, speaker/listener’s unique characteristics (individual differences), and language’s sound shape that influences the aesthetic rating. We deliberately wanted to address the question of the musical allure of the widely spoken languages that are prototypically (through public surveys and on online forums) declared the most melodious (e.g., Italian). Unfortunately, these languages are also common languages learned in schools and they enjoy public familiarity. In our previous study (Reiterer et al., 2020) we measured the share that familiarity contributes to the overall aesthetic judgments and came to the conclusion that although it plays a prominent role (about 40% of variance), there is more to the story. The present study was dedicated to the acoustic-phonetic dimension only without an explicit and further investigation of familiarity. For future studies, the strategy could be to use only unfamiliar languages or artificial languages or focus on non-European listeners.

Also, it would be beneficial to incorporate a variety of languages in future studies, preferably of languages that belong to different families and show a range of phonetic and acoustic features. The present study was a first exploration into aesthetic preferences for the sound of only 16 European languages.

## Data Availability Statement

The raw data supporting the conclusions of this article will be made available by the authors, without undue reservation.

## Ethics Statement

Ethical review and approval was not required for the study on human participants in accordance with the local legislation and institutional requirements. The patients/participants provided their written informed consent to participate in this study.

## Author Contributions

SR contributed to conception and experimental design of the scientific work. SR and VK involved in processing of data collection, contributed to data analysis and interpretation, drafted, and wrote and revised the article critically. Both authors contributed to the article and approved the submitted version.

## Conflict of Interest

The authors declare that the research was conducted in the absence of any commercial or financial relationships that could be construed as a potential conflict of interest.
