# Supplementary material for: Eros, Beauty, and Phon-Aesthetic Judgements of Language Sound. We Like It Flat and Fast, but Not Melodious. Comparing Phonetic and Acoustic Features of 16 European Languages
Source: Front Hum Neurosci. 2021 Feb 23;15:578594. doi: 10.3389/fnhum.2021.578594 (PMC7940689; doi:10.3389/fnhum.2021.578594)
Supplement: Supplementary file 1 [file Data_Sheet_1.docx]

**Supplemental materials**

Table 1S. Aesthetic ratings for all languages.

| Table 1S |  | Basque | **Catalan** | **Croatian** | **Danish** | **English** | **French** | **German** | **Greek** |
| --- | --- | --- | --- | --- | --- | --- | --- | --- | --- |
| N= |  | 45 | 45 | 45 | 45 | 45 | 45 | 45 | 45 |
| missing |  | 0 | 0 | 0 | 0 | 0 | 0 | 0 | 0 |
| Mean | **Grand Avg** | 51,36 | 58,21 | 52,06 | 48,3 | 68,94 | 71,83 | 49,22 | 48,01 |
| Median | **Grand Avg** | 50,68 | 55,90 | 54,50 | 48,81 | 69,54 | 74,04 | 50,40 | 47,22 |
| SD +/- | **Grand Avg** | 10,90 | 12,61 | 13,52 | 10,20 | 11,80 | 12,44 | 11,36 | 13,90 |
| Min | **Grand Avg** | 31,73 | 33,05 | 25,73 | 27,77 | 46,55 | 40,59 | 24,95 | 25,55 |
| Max | **Grand Avg** | 72,55 | 83,18 | 78,45 | 66,82 | 95,55 | 97,32 | 76,00 | 77,73 |
|  |  | **Hungarian** | **Icelandic** | **Italian** | **Polish** | **Russian** | **Spanish** | **Ukrainian** | **Welsh** |
| N= |  | 45 | 45 | 45 | 45 | 45 | 45 | 45 | 45 |
| missing |  | 0 | 0 | 0 | 0 | 0 | 0 | 0 | 0 |
| Mean | **Grand Avg** | 46,08 | 52,91 | 63,33 | 44,71 | 50,45 | 62,29 | 49,72 | 41,93 |
| Median | **Grand Avg** | 45,54 | 50,50 | 64,50 | 46,59 | 50,59 | 63,00 | 48,72 | 43,54 |
| SD +/- | **Grand Avg** | 13,12 | 14,00 | 15,19 | 11,43 | 14,14 | 13,74 | 14,71 | 12,81 |
| Min | **Grand Avg** | 20,09 | 23,91 | 19,55 | 17,73 | 26,23 | 25,55 | 19,77 | 14,59 |
| Max | **Grand Avg** | 82,50 | 85,64 | 97,77 | 72,73 | 88,23 | 90,23 | 88,09 | 68,55 |
|  |  |  |  |  |  |  |  |  |  |
|  |  | **Basque** | **Catalan** | **Croatian** | **Danish** | **English** | **French** | **German** | **Greek** |
| N= |  | 45 | 45 | 45 | 45 | 45 | 45 | 45 | 45 |
| missing |  | 0 | 0 | 0 | 0 | 0 | 0 | 0 | 0 |
| Mean | **BEAUTY** | 54,05 | 62,30 | 58,90 | 51,14 | 70,07 | 70,24 | 47,43 | 50,78 |
| Median | **BEAUTY** | 54,28 | 61,57 | 59,71 | 51,71 | 70,42 | 72,57 | 49,42 | 51,57 |
| SD +/- | **BEAUTY** | 14,45 | 14,44 | 17,50 | 13,42 | 13,12 | 15,04 | 15,04 | 14,36 |
| Min | **BEAUTY** | 25,14 | 31,71 | 26,00 | 19,71 | 41,29 | 28,29 | 19,57 | 25,43 |
| Max | **BEAUTY** | 90,71 | 90,57 | 97,14 | 77,29 | 98,86 | 100,00 | 84,29 | 81,14 |
|  |  | **Hungarian** | **Icelandic** | **Italian** | **Polish** | **Russian** | **Spanish** | **Ukrainian** | **Welsh** |
| N= |  | 45 | 45 | 45 | 45 | 45 | 45 | 45 | 45 |
| missing |  | 0 | 0 | 0 | 0 | 0 | 0 | 0 | 0 |
| Mean | **BEAUTY** | 51,73 | 55,60 | 67,49 | 49,33 | 53,10 | 66,72 | 53,51 | 44,30 |
| Median | **BEAUTY** | 52,42 | 51,5714 | 71,42 | 49,14 | 50,85 | 66,71 | 54,14 | 43,71 |
| SD +/- | **BEAUTY** | 16,09 | 17,49 | 17,07 | 15,13 | 15,59 | 15,82 | 15,63 | 18,06 |
| Min | **BEAUTY** | 15,14 | 3,29 | 19,14 | 10,71 | 27,29 | 30,86 | 24,43 | ,00 |
| Max | **BEAUTY** | 93,00 | 89,00 | 98,71 | 74,29 | 90,00 | 100,00 | 86,29 | 89,14 |
|  |  |  |  |  |  |  |  |  |  |
|  |  | **Basque** | **Catalan** | **Croatian** | **Danish** | **English** | **French** | **German** | **Greek** |
| N= |  | 45 | 45 | 45 | 45 | 45 | 45 | 45 | 45 |
| missing |  | 0 | 0 | 0 | 0 | 0 | 0 | 0 | 0 |
| Mean | **STATUS** | 50,34 | 55,26 | 49,86 | 55,38 | 75,97 | 74,87 | 62,26 | 49,70 |
| Median | **STATUS** | 50,62 | 55,12 | 49,87 | 53,50 | 74,62 | 76,87 | 61,12 | 47,75 |
| SD +/- | **STATUS** | 12,59 | 13,12 | 13,78 | 12,64 | 12,81 | 12,60 | 12,84 | 15,62 |
| Min | **STATUS** | 18,63 | 32,13 | 19,38 | 26,38 | 51,00 | 50,38 | 27,38 | 20,88 |
| Max | **STATUS** | 75,75 | 80,38 | 81,25 | 86,13 | 100,00 | 100,00 | 94,38 | 88,88 |
|  |  | **Hungarian** | **Icelandic** | **Italian** | **Polish** | **Russian** | **Spanish** | **Ukrainian** | **Welsh** |
| N= |  | 45 | 45 | 45 | 45 | 45 | 45 | 45 | 45 |
| missing |  | 0 | 0 | 0 | 0 | 0 | 0 | 0 | 0 |
| Mean | **STATUS** | 47,19 | 57,17 | 62,34 | 45,85 | 54,33 | 61,39 | 51,76 | 45,62 |
| Median | **STATUS** | 47,37 | 57,00 | 62,75 | 46,87 | 53,37 | 59,75 | 51,37 | 46,37 |
| SD +/- | **STATUS** | 14,18 | 16,38 | 14,55 | 11,41 | 17,45 | 13,38 | 16,22 | 13,60 |
| Min | **STATUS** | 22,88 | 16,75 | 20,13 | 19,50 | 12,88 | 28,63 | 19,38 | 7,25 |
| Max | **STATUS** | 78,88 | 84,88 | 96,25 | 76,25 | 94,88 | 89,88 | 92,25 | 69,38 |
|  |  |  |  |  |  |  |  |  |  |
|  |  | **Basque** | **Catalan** | **Croatian** | **Danish** | **English** | **French** | **German** | **Greek** |
| N= |  | 45 | 45 | 45 | 45 | 45 | 45 | 45 | 45 |
| missing |  | 0 | 0 | 0 | 0 | 0 | 0 | 0 | 0 |
| Mean | **EROS** | 51,11 | 55,86 | 45,00 | 32,75 | 53,38 | 71,54 | 32,41 | 41,06 |
| Median | **EROS** | 57,50 | 56,50 | 42,75 | 31,75 | 55,75 | 77,50 | 34,50 | 38,50 |
| SD +/- | **EROS** | 17,28 | 18,88 | 18,82 | 16,84 | 20,26 | 21,30 | 19,61 | 20,65 |
| Min | **EROS** | 10,50 | 6,25 | 5,50 | ,00 | 3,50 | 10,25 | ,00 | 1,50 |
| Max | **EROS** | 76,50 | 100,00 | 75,75 | 67,00 | 89,75 | 100,00 | 81,50 | 82,75 |
|  |  | **Hungarian** | **Icelandic** | **Italian** | **Polish** | **Russian** | **Spanish** | **Ukrainian** | **Welsh** |
| N= |  | 45 | 45 | 45 | 45 | 45 | 45 | 45 | 45 |
| missing |  | 0 | 0 | 0 | 0 | 0 | 0 | 0 | 0 |
| Mean | **EROS** | 32,79 | 41,50 | 59,23 | 33,66 | 40,83 | 59,79 | 38,32 | 31,68 |
| Median | **EROS** | 32,75 | 42,75 | 64,75 | 34,00 | 40,00 | 61,75 | 37,00 | 35,25 |
| SD +/- | **EROS** | 18,55 | 18,96 | 21,97 | 15,93 | 18,77 | 21,89 | 21,11 | 18,18 |
| Min | **EROS** | 2,50 | ,00 | ,00 | 6,50 | 5,00 | 5,75 | 2,25 | ,75 |
| Max | **EROS** | 79,50 | 85,00 | 100,00 | 67,00 | 87,75 | 100,00 | 90,00 | 69,75 |
|  |  |  |  |  |  |  |  |  |  |
|  |  | **Basque** | **Catalan** | **Croatian** | **Danish** | **English** | **French** | **German** | **Greek** |
| N= |  | 45 | 45 | 45 | 45 | 45 | 45 | 45 | 45 |
| missing |  | 0 | 0 | 0 | 0 | 0 | 0 | 0 | 0 |
| Mean | **Softness** | 51,31 | 63,30 | 51,34 | 40,55 | 65,35 | 75,13 | 25,44 | 47,72 |
| Median | **Softness** | 50,00 | 65,00 | 51,00 | 40,00 | 64,50 | 73,50 | 22,50 | 46,50 |
| SD +/- | **Softness** | 16,38 | 18,30 | 18,41 | 16,28 | 15,99 | 12,62 | 16,80 | 16,28 |
| Min | **Softness** | 25,00 | 5,50 | 6,50 | 2,50 | 28,00 | 33,00 | ,50 | 17,00 |
| Max | **Softness** | 84,50 | 100,00 | 98,00 | 80,00 | 98,00 | 100,00 | 64,50 | 84,50 |
|  |  | **Hungarian** | **Icelandic** | **Italian** | **Polish** | **Russian** | **Spanish** | **Ukrainian** | **Welsh** |
| N= |  | 45 | 45 | 45 | 45 | 45 | 45 | 45 | 45 |
| missing |  | 0 | 0 | 0 | 0 | 0 | 0 | 0 | 0 |
| Mean | **Softness** | 47,40 | 45,91 | 65,32 | 42,91 | 40,56 | 60,06 | 45,44 | 38,10 |
| Median | **Softness** | 45,00 | 45,00 | 67,00 | 45,50 | 35,00 | 65,00 | 44,50 | 35,50 |
| SD +/- | **Softness** | 16,68 | 16,57 | 17,61 | 18,75 | 19,86 | 19,86 | 19,16 | 15,39 |
| Min | **Softness** | 16,00 | 16,00 | 27,00 | 3,00 | 2,00 | 18,00 | 9,50 | 6,50 |
| Max | **Softness** | 80,00 | 91,00 | 100,00 | 81,00 | 79,00 | 100,00 | 88,00 | 74,50 |
|  |  |  |  |  |  |  |  |  |  |
|  |  | **Basque** | **Catalan** | **Croatian** | **Danish** | **English** | **French** | **German** | **Greek** |
| N= |  | 45 | 45 | 45 | 45 | 45 | 45 | 45 | 45 |
| missing |  | 0 | 0 | 0 | 0 | 0 | 0 | 0 | 0 |
| Mean | **Orderliness** | 41,82 | 52,31 | 51,47 | 50,78 | 74,16 | 53,13 | 72,13 | 43,53 |
| Median | **Orderliness** | 38,00 | 60,00 | 55,00 | 55,00 | 75,00 | 58,00 | 73,00 | 38,00 |
| SD +/- | **Orderliness** | 20,7 | 22,80 | 20,28 | 22,22 | 16,32 | 22,36 | 19,51 | 21,26 |
| Min | **Orderliness** | 0 | 7 | 0 | 0 | 39 | 4 | 20 | 0 |
| Max | **Orderliness** | 80 | 93 | 100 | 97 | 100 | 88 | 100 | 88 |
|  |  | **Hungarian** | **Icelandic** | **Italian** | **Polish** | **Russian** | **Spanish** | **Ukrainian** | **Welsh** |
| N= |  | 45 | 45 | 45 | 45 | 45 | 45 | 45 | 45 |
| missing |  | 0 | 0 | 0 | 0 | 0 | 0 | 0 | 0 |
| Mean | **Orderliness** | 48,24 | 59,64 | 54,69 | 51,16 | 59,09 | 52,93 | 61,09 | 44,42 |
| Median | **Orderliness** | 46,00 | 63,00 | 62,00 | 47,00 | 62,00 | 56,00 | 62,00 | 41,00 |
| SD +/- | **Orderliness** | 24,11 | 21,03 | 23,16 | 21,73 | 21,89 | 21,66 | 19,59 | 21,91 |
| Min | **Orderliness** | 10 | 4 | 13 | 4 | 7 | 6 | 14 | 0 |
| Max | **Orderliness** | 97 | 100 | 93 | 96 | 100 | 99 | 100 | 81 |

Table 2S: Information about the experiment’s languages

| **Language** | **Language family** | **Recording source** | **Speaker’s gender** | **Sampling rate** | **Length (s)** |
| --- | --- | --- | --- | --- | --- |
| Basque | Other (Basque) | Hualde, Lujanbio, & Zubiri (2010) | female | 44100 Hz, 16bit, Mono | 40 |
| Catalan | Romance | Carbonell & Llisterri (1992) | male | 16000 Hz, 16bit, Mono | 31 |
| Croatian | Slavic | Landau, Lončarić, Horga, & Škarić (1995) | female | 16000 Hz, 16bit, Mono | 40 |
| Danish | Germanic | Grønnum (1998) | female | 44100 Hz, 16bit, Mono | 30 |
| English | Germanic | Roach (2004) | female | 22050 Hz, 16bit, Mono | 37 |
| French | Romance | Fougeron & Smith (1993) | female | 22050 Hz, 16bit, Mono | 33 |
| German | Germanic | Abercombie (2013) | male | 48000 Hz, 16bit, Mono | 44 |
| Greek | Other (Hellenic) | Verhoeven (2019) | male | 22050 Hz, 16bit, Mono | 39 |
| Hungarian | Other (Finno-Ugric) | Szende (1994) | male | 16000 Hz, 16bit, Mono | 40 |
| Icelandic | Germanic | Abercombie (2013) | male | 48000 Hz, 16bit, Mono | 33 |
| Italian | Romance | Private recording | female | 44100 Hz, 16bit, Mono | 43 |
| Polish | Slavic | Jassem (2003) | female | 16000 Hz, 16bit, Mono | 40 |
| Russian | Slavic | Yanushevskaya & Bunčić (2015) | male | 44100 Hz, 16bit, Mono | 37 |
| Spanish | Romance | Martínez-Celdrán, Fernández-Planas, & Carrera-Sabaté (2003) | female | 22050 Hz, 16bit, Mono | 34 |
| Ukrainian | Slavic | Pompino-Marschall, Steriopolo, & Żygis (2017) | male | 44100 Hz, 16bit, Mono | 44 |
| Welsh | Other (Celtic) | Abercombie (2013) | male | 48000 Hz, 16bit, Mono | 40 |

| table S3 | L1 community size | Learned as Lx | Recognition rate | Speech rate set 1 | F0 | BEAUTY | STATUS | EROS | SOFTNESS | ORDERLI-  NESS | melody | Melody variance set 1 | Text length set 1 | Speech rate set 2 | Melody variance set 2 | Text length set 2 |
| --- | --- | --- | --- | --- | --- | --- | --- | --- | --- | --- | --- | --- | --- | --- | --- | --- |
| L1 community size | \| 1 \| \| --- \| |  |  |  |  |  |  |  |  |  |  |  |  |  |  |  |
| Learned as Lx | \| **0.69*** \| \| --- \| | \| 1 \| \| --- \| |  |  |  |  |  |  |  |  |  |  |  |  |  |  |
| Recognition rate | \| **0.86**** \| \| --- \| | \| **0.91**** \| \| --- \| | \| 1 \| \| --- \| |  |  |  |  |  |  |  |  |  |  |  |  |  |
| Speech rate set 1 | \| -0.1 \| \| --- \| | \| -0.04 \| \| --- \| | \| -0.02 \| \| --- \| | \| 1 \| \| --- \| |  |  |  |  |  |  |  |  |  |  |  |  |
| F0 | \| 0.15 \| \| --- \| | \| 0.28 \| \| --- \| | \| 0.21 \| \| --- \| | \| -0.03 \| \| --- \| | \| 1 \| \| --- \| |  |  |  |  |  |  |  |  |  |  |  |
| BEAUTY | \| 0.23 \| \| --- \| | \| 0.41 \| \| --- \| | \| 0.37 \| \| --- \| | \| **0.71**** \| \| --- \| | \| 0.2 \| \| --- \| | \| 1 \| \| --- \| |  |  |  |  |  |  |  |  |  |  |
| STATUS | \| **0.46^t^** \| \| --- \| | \| **0.6**** \| \| --- \| | \| **0.55*** \| \| --- \| | \| 0.36 \| \| --- \| | \| 0.16 \| \| --- \| | \| **0.68**** \| \| --- \| | \| 1 \| \| --- \| |  |  |  |  |  |  |  |  |  |
| EROS | \| 0.2 \| \| --- \| | \| 0.3 \| \| --- \| | \| 0.32 \| \| --- \| | \| **0.81**** \| \| --- \| | \| 0.2 \| \| --- \| | \| **0.92**** \| \| --- \| | \| **0.57*** \| \| --- \| | \| 1 \| \| --- \| |  |  |  |  |  |  |  |  |
| SOFTNESS | \| 0.15 \| \| --- \| | \| 0.33 \| \| --- \| | \| 0.36 \| \| --- \| | \| **0.64**** \| \| --- \| | \| 0.25 \| \| --- \| | \| **0.91**** \| \| --- \| | \| **0.48^t^** \| \| --- \| | \| **0.92**** \| \| --- \| | \| 1 \| \| --- \| |  |  |  |  |  |  |  |
| ORDERLI-NESS | \| **0.6**** \| \| --- \| | \| **0.52*** \| \| --- \| | \| **0.55*** \| \| --- \| | \| 0.05 \| \| --- \| | \| -0.11 \| \| --- \| | \| 0.39 \| \| --- \| | \| **0.71**** \| \| --- \| | \| 0.19 \| \| --- \| | \| 0.11 \| \| --- \| | \| 1 \| \| --- \| |  |  |  |  |  |  |
| melody | \| 0.16 \| \| --- \| | \| 0.39 \| \| --- \| | \| 0.35 \| \| --- \| | \| **0.69**** \| \| --- \| | \| 0.3 \| \| --- \| | \| **0.95**** \| \| --- \| | \| **0.57**** \| \| --- \| | \| **0.85**** \| \| --- \| | \| **0.89**** \| \| --- \| | \| 0.27 \| \| --- \| | \| 1 \| \| --- \| |  |  |  |  |  |
| Melody variance set 1 | \| 0.15 \| \| --- \| | \| 0.07 \| \| --- \| | \| 0.1 \| \| --- \| | \| **-0.61*** \| \| --- \| | \| 0.21 \| \| --- \| | \| **-0.43^t^** \| \| --- \| | \| -0.19 \| \| --- \| | \| **-0.52*** \| \| --- \| | \| -0.41 \| \| --- \| | \| 0.15 \| \| --- \| | \| -0.33 \| \| --- \| | \| 1 \| \| --- \| |  |  |  |  |
| Text length set 1 | \| 0.2 \| \| --- \| | \| 0.18 \| \| --- \| | \| 0.2 \| \| --- \| | \| -0.37 \| \| --- \| | \| 0.02 \| \| --- \| | \| -0.34 \| \| --- \| | \| -0.3 \| \| --- \| | \| -0.35 \| \| --- \| | \| -0.26 \| \| --- \| | \| 0.04 \| \| --- \| | \| -0.3 \| \| --- \| | \| **0.44^t^** \| \| --- \| | \| 1 \| \| --- \| |  |  |  |
| Speech rate set 2 | \| -0.12 \| \| --- \| | \| 0.02 \| \| --- \| | \| -0.02 \| \| --- \| | \| **0.79**** \| \| --- \| | \| 0.03 \| \| --- \| | \| **0.52*** \| \| --- \| | \| 0.18 \| \| --- \| | \| **0.69**** \| \| --- \| | \| **0.57*** \| \| --- \| | \| -0.24 \| \| --- \| | \| **0.53*** \| \| --- \| | \| **-0.54*** \| \| --- \| | \| -0.3 \| \| --- \| | \| 1 \| \| --- \| |  |  |
| Melody variance set 2 | \| 0 \| \| --- \| \|  \| | \| -0.2 \| \| --- \| \|  \| | \| -0.09 \| \| --- \| \|  \| | \| **-0.57*** \| \| --- \| \|  \| | \| -0.37 \| \| --- \| \|  \| | \| **-0.57*** \| \| --- \| \|  \| | \| -0.21 \| \| --- \| | \| **-0.61**** \| \| --- \| \|  \| | \| **-0.59*** \| \| --- \| \|  \| | \| -0.01 \| \| --- \| \|  \| | \| **-0.53*** \| \| --- \| \|  \| | \| 0.4 \| \| --- \| \|  \| | \| **0.42^t^** \| \| --- \| \|  \| | \| **-0.63**** \| \| --- \| \|  \| | \| 1 \| \| --- \| |  |
| Text length set 2 | -0.07 | -0.15 | -0.01 | -0.35 | -0.1 | -0.39 | \| -0.09 \| \| --- \| | -0.37 | -0.24 | 0.02 | -0.33 | **0.6*** | **0.46^t^** | -0.34 | **0.6*** | 1 |

Table S3 Supplementary material – Ad Fig 7 in the manuscript: overview table of all correlation coefficients (Spearman’s Rho) of all zero order correlations.

**p <0.1ᵗ (trend) p <0.05* (significant) p < 0.01** (highly significant)**

table S3


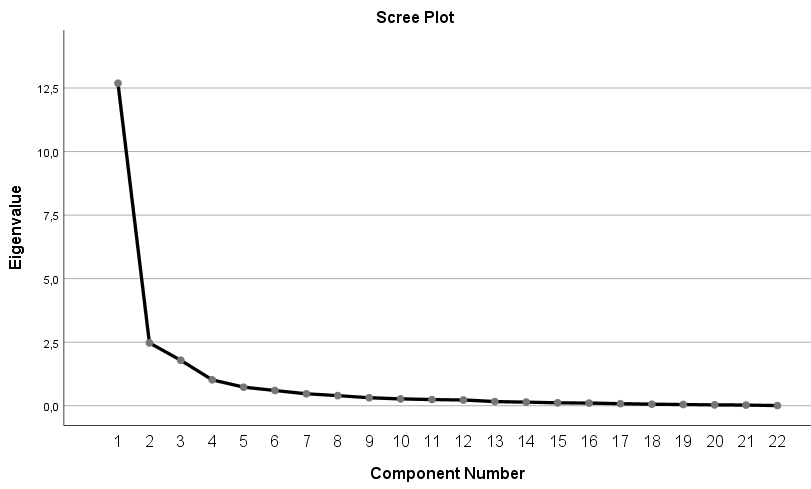
Fig 1S


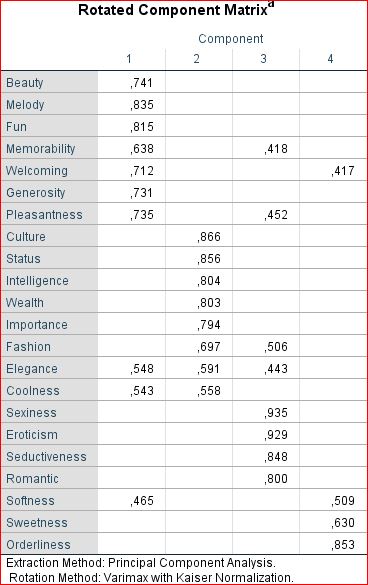


Figures 1S+2S. The exploratory factor analysis (EFA: described in Reiterer et al., 2020); that resulted in the reduction of 22 aesthetic ratings to five factors based on N=45.

**Qualitative data**

*Note: Since only 15% of all participants used the option of giving associative comments, these data should be interpreted with caution and only be seen as a source for additional information / ideas / inspiration. The responses are not proof-read for typos and misspellings.*

***Free comments about Welsh:***

He sounds happy

The speaker's voice sounds like the person is somebody interested in something

As for me~ it sounds like half English~ half Indian~ Half Bulgarian and Hungarian

It sounds very exotic to me compared to all the other languages.

I find it slightly repellent~ because it sounds like the speaker would have mouth full of saliva.

The pronunciation seeems very hard

So many back/uvular sounds...

Comparing this track to the German one, which also had a male voice, this one sounds nicer.

I spent a year in Wales and may have some strong (positive) emotions towards this language

***Free comments about Ukrainian:***

The speaker has a nice~ calm~ happy voice-a mix

A little too loud. It feels like the speaker speaks directly to a microphone.

A good bass voice

Russian is also one of my favorite languages~ pronunciation-wise. I didn't find the recording/voice to be the best but I generally really enjoy listening to Russian.

It seems like the speaker is trying very hard to read slowly

The speakers voice sound sympathic to me.

Interesting rather soft consonants and lots of vowels make the language sound softer

The speaker seems very calm.

***Free comments about Spanish:***

I find it pleasant to listen to but not particularly interesting

I think the speakers voice is very neutral. But their cadence is more pleasant than unpleasant.

To me it sounds rounded referring to a shape. Instead of sweet (as opposite to harsh) I would select rounded in this case as opposite to harsh (or even better sharp).

Probably because of the positive associations with Spain and Spanish people (less so because of language-specific characteristics)

Easy to follow~ strong and convincing voice

She seemed like a decisive person

***Free comments about Russian:***

As a person that uses russian language almost daily, I can't concentrate on the voice. I automatically concentrate on the meaning

A normal and pleasant voice.

The speaker sounded less warm than the Romance speaker [Catalan].

***Free comments about Polish:***

The women speaker seems like she is middle age

A bit lound and strong with stressing some words

Neutral voice

I find it interesting but it's not a language I would feel particularly attracted to. By the way, now that I hear it for a third time~ I believe it's Polish ("-niego" ending)

I find it difficult to rate languages in most categories~ but I feel I find it even harder for Slavic languages.

Her voice sounds pleasant

***Free comments about Italian:***

Relaxing and pleasant voice

Italian is my mothertongue so probably it influenced my opinions

recording was bad~ with reverberation.

Italian is my favorite Romance language (together with my L1 Portuguese) and I find the speaker's voice very pleasant to listen to as well.

Open syllables make it seem very pleasant for me

The language sounds for me very emotional and prosodic.

Rather slow speaking, average language speed higher, easy to understand, pleasant flow

It's a female voice, so in comparison to the male voice for the German track, it sounds softer.

***Free comments about Icelandic:***

I find it pleasant to listen to and it sounds very high-cultured.

The speaker's voice seems very soft.

Finnish?

The speaker sounds proud.

Funny language

***Free comments about Hungarian:***

Hungarian is in my opinion one of the most melodic and logical languages in the world. I can understand it to a B2 level. However, since I no longer use the language in daily life, it is a little bit rusty. I find it extremely pleasant to listen to.

Hungarian

very warm and nice voice. It’s also a nice rhythm.

Seems like the speaker has a slight speech impediment (pronunciation of sibilants).

***Free comments about Greek:***

Talks without passion

I've learned Modern Greek for about 5 months and enjoy listening to it.

To me it sounds like the speaker is boringly reading some text. I cannot really say the language itself sounds boring to me.

Some aspects would be best described as "softened harshness". The consonants are nicely connected in the flow of the language.

Feels like summer

Very nice and pleasant voice

Slow, nice and relaxing melodic

The speaker sounded smart

The sound quality was worse than in the other samples

The voice seems monotonous, as if the speaker was bored or tired.

***Free comments about German:***

A bit too fast, but still pleasant, sometimes strong with stressing some words

Pronunciation is too hard for my taste as native speaker

I speak German as a Second Language and I enjoy how orderly it sounds.

Harsh

Austrian German?

German used to sound very aggressive to me before I learned it. The more I learned German, the more positive emotions I would attach to it. However, I prefer the Austrian German over German German.

It seems like a male voice.

The pronunciation sounds strange to me

***Free comments about French:***

Beautiful

“Veery” pleasant and super soft

I have mixed feelings about French. I like it depending on the speaker, but I do not find it particularly charming, romantic or beautiful. I can understand and speak the language but it's not my favourite Romance language.

Again calming and in this case I experienced slight ASMR. [Autonomous Sensory Meridian Response, kind of “chill feeling”]

French sounds to me very soft and "round". It is often the language of love and in many songs some phrases are in French. (Lady Gaga: Bad Romance / Christina Aguilera : Lady Marmalade / ABBA: Voulez-vous)

wunderschön gesprochen

French

The speaker seems calm, very pleasant.

***Free comments about English:***

Exact pronunciation

English is still my best foreign language. Although, I tend to speak with a more American accent. I really enjoy hearing British accents too.

Initially I could not help not to judge the language including the meaning. The experience of the sound immediately gave me visuals and the atmosphere. Most of the categories/pairs do not make sense to me - maybe some of them would have sense for judging music.

Probably pleasant because it's so familiar

Good pronouncing

I liked the voice of the speaker and I prefer UK English over American / Australian / New Zealand / African English

A female speaking in British English this way seems like an educated person

The speaker sounded sophisticated and educated, if a bit unapproachable.

***Free comments about Danish:***

Too fast

I have "flirted" with Scandinavian languages but never learned any long enough. I find them generally pleasant and interesting from the perspective of a L2 German/English speaker (Germanic roots become apparent despite the different pronunciation). I also find the pronunciation pleasant to listen to.

Again, I find the speaker's voice calming.

Interesting flow. Some words recognised from English

In comparison to the Italian track (had a female voice), this one sounded less nice, but I also think the speaker in this track seemed older.

***Free comments about Croatian:***

The speaker is a calm person

A little too fast. It would be better with making a few pauses

Again, I find it interesting compared to Russian (a Slavic language that I've been learning for the past hour) and it sounds better than the last recording (Polish?) but again, I think there are better sounding Slavic languages.

The speaker voice is very well articulated. The harsh Slavic noises are often softened.

She doesn’t make stops/pauses between sentences.

I associate this language (BKS) with holidays in Croatia and emotional music, so it is pleasant to me.

The speaker sounds quite old and wise

***Free comments about Catalan:***

Soft and pleasant

It sounds like a Spanish dialect

It's very similar to Spanish (a language I understand and speak). But it has an interesting touch with what initially seem "irregularities". I find it very pleasant and welcoming to listen to.

The recording sounds calming to me and it would initiate an ASMR response if the speaker would speak more quietly.

So many fricatives

Speaks too fast. Doesn't enunciate as clearly as other examples

Melodic

The speaker seemed like a warm person

***Free comments about Basque:***

The woman seems like she is aged between 25-35.

Pleasant voice

It's pleasant to listen to but I don't know how to feel about the language.

It sounds a bit broken to me - I am not sure if the language sounds like that (to me) or is it just how speaker was reading/saying the text (having many stop/braking points).

The consonants are harsh and the vowels are pronounced very quickly

Rather nice voice, could imagine that it sounds even sexy or seductive to opposite sex people. Nice language in general.

*Basque was one of the most confusing languages to guess. Below is the list of the responses that the participants gave to answer the question “What language is this?”:

Portugese

Catalan

Latvian

Romance language - Spanish?

Something between spanish-mexican-argentinian, difficult to say

Romanic languag

Portuguese

Spanish

Spanisch

I don't recognize it

Russian

Romanski

Not familiar

I couldn't understand the language but if I had to guess, I would say it's Romanian.

Romance

Basque

Portugal?

Romance

Romance

Ugrofinska jezikovna skupina

Slavic?

Spanish

Romance language

Maltese?

No idea

Something like Spanish

First, I thought it was Romanian~ but then it sounded more like Greek.

Keine Ahnung [No idea].

Portuguese

Some Romance and Slavic elements, proably Slowenic?

Portugal

Maybe Turkish

Catalan

Basque

Romance language

Catalan

Romanian?

Something romanic?

Catalan

Ugrofinski?

Ladin or another romance language

Something similar to Spanish

I THINK it's Portugese.

Don't know

Portuguese

***Free comments about Doublet recording:***

This measure was *a random replication of one of the languages repeated again for reliability purposes*. *Unfortunately, only three out of 45 participants responded to this question and only one person suspected, that they might have already heard the language before. The original and the repeated recording (doublet) correlated significantly in terms of the aesthetic ratings.*

I think I already heard this recording?

She seemed like a decisive person

Very warm voice. The language sounds funny.
